# Supplementary material for: Changing risk factors for developing SARS-CoV-2 infection from Delta to Omicron
Source: PLoS One. 2024 May 15;19(5):e0299714. doi: 10.1371/journal.pone.0299714 (PMC11095668; doi:10.1371/journal.pone.0299714)

Supplementary file

Changing risk factors for developing SARS-CoV-2 infection from Delta to Omicron

Paul R. Hunter MD, Julii Brainard PhD

Further analyses of risk factors not significant for trend over time

Gender Female v Male

Time period	Risk Difference	SE	Approximate 95% CI		
1	-0.05	0.04	-0.128399	0.028399	21 November 2021 to 04 December 2021
2	-0.08	0.04	-0.158399	-0.001601	05 December 2021 to 18 December 2021
3	-0.12	0.03	-0.178799	-0.061201	19 December 2021 to 01 January 2022
4	-0.03	0.02	-0.069199	0.009199	02 January 2022 to 15 January 2022
5	0.01	0.02	-0.029199	0.049199	16 January 2022 to 29 January 2022
6	-0.03	0.02	-0.069199	0.009199	30 January 2022 to 12 February 2022
7	-0.07	0.03	-0.128799	-0.011201	13 February 2022 to 26 February 2022
8	-0.05	0.02	-0.089199	-0.010801	27 February 2022 to 12 March 2022
9	-0.04	0.02	-0.079199	-0.000801	13 March 2022 to 26 March 2022
10	-0.07	0.02	-0.109199	-0.030801	27 March 2022 to 09 April 2022
11	-0.05	0.02	-0.089199	-0.010801	10 April 2022 to 23 April 2022
12	-0.09	0.03	-0.148799	-0.031201	24 April 2022 to 07 May 2022

Non-combinability of studies

Cochran Q = 20.306189 (df = 11) $P = 0.0413$
Moment-based estimate of between studies variance = 0.000468
I_2 (inconsistency) = 45.8% (95% CI = 0% to 70.8%)

Random effects (DerSimonian-Laird)

Pooled risk difference = -0.05052 (95% CI = -0.069016 to -0.032023)
Z (test Risk Difference) = -5.35327 $P < 0.0001$

Summary meta-analysis plot [random effects]

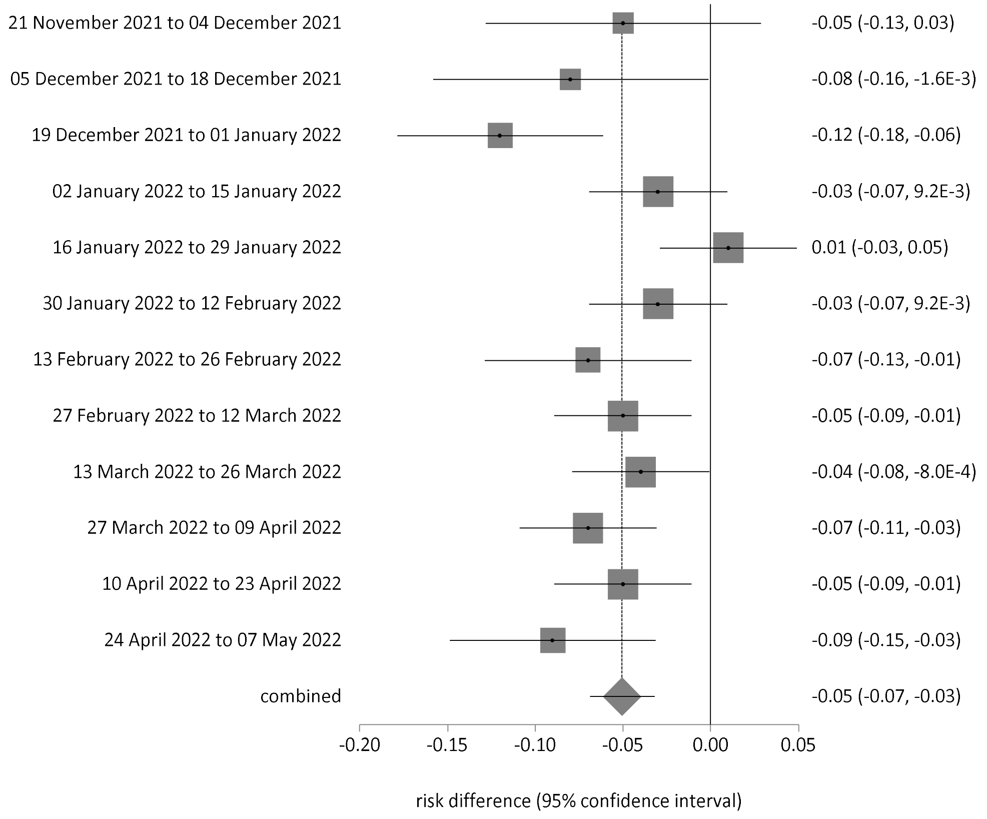

Household size=3 v size =1

Time period	Risk Difference	SE	Approximate 95% CI		
1	0.28	0.09	0.103603	0.456397	21 November 2021 to 04 December 2021
2	0.23	0.08	0.073203	0.386797	05 December 2021 to 18 December 2021
3	0	0.05	-0.097998	0.097998	19 December 2021 to 01 January 2022
4	0.12	0.04	0.041601	0.198399	02 January 2022 to 15 January 2022
5	0.36	0.05	0.262002	0.457998	16 January 2022 to 29 January 2022
6	0.36	0.05	0.262002	0.457998	30 January 2022 to 12 February 2022
7	0.03	0.05	-0.067998	0.127998	13 February 2022 to 26 February 2022
8	0.26	0.05	0.162002	0.357998	27 February 2022 to 12 March 2022
9	0.24	0.04	0.161601	0.318399	13 March 2022 to 26 March 2022
10	0.2	0.04	0.121601	0.278399	27 March 2022 to 09 April 2022
11	0.24	0.05	0.142002	0.337998	10 April 2022 to 23 April 2022
12	0.2	0.06	0.082402	0.317598	24 April 2022 to 07 May 2022

Non-combinability of studies

Cochran Q = 56.03182 (df = 11) P < 0.0001
Moment-based estimate of between studies variance = 0.010311
I_2 (inconsistency) = 80.4% (95% CI = 64.6% to 87.3%)

Random effects (DerSimonian-Laird)

Pooled risk difference = 0.207377 (95% CI = 0.142269 to 0.272484)
Z (test Risk Difference) = 6.242777 P < 0.0001

Summary meta-analysis plot [random effects]

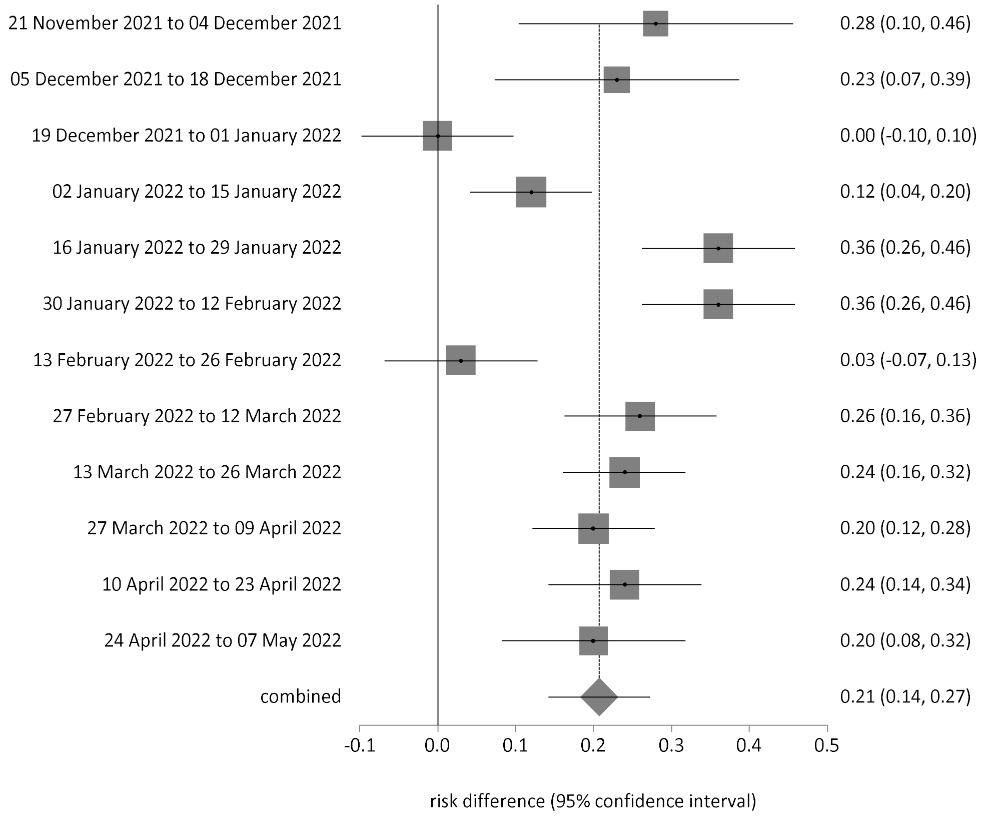

Household size=4 v size=1

Time period	Risk Difference	SE	Approximate 95% CI		
1	0.43	0.09	0.253603	0.606397	21 November 2021 to 04 December 2021
2	0.44	0.07	0.302803	0.577197	05 December 2021 to 18 December 2021
3	0.09	0.05	-0.007998	0.187998	19 December 2021 to 01 January 2022
4	0.11	0.04	0.031601	0.188399	02 January 2022 to 15 January 2022
5	0.49	0.05	0.392002	0.587998	16 January 2022 to 29 January 2022
6	0.45	0.05	0.352002	0.547998	30 January 2022 to 12 February 2022
7	0.17	0.05	0.072002	0.267998	13 February 2022 to 26 February 2022
8	0.27	0.05	0.172002	0.367998	27 February 2022 to 12 March 2022
9	0.24	0.04	0.161601	0.318399	13 March 2022 to 26 March 2022
10	0.23	0.04	0.151601	0.308399	27 March 2022 to 09 April 2022
11	0.22	0.05	0.122002	0.317998	10 April 2022 to 23 April 2022
12	0.05	0.07	-0.087197	0.187197	24 April 2022 to 07 May 2022

Non-combinability of studies

Cochran Q = 84.432021 (df = 11) $P < 0.0001$
Moment-based estimate of between studies variance = 0.01691
I_2 (inconsistency) = 87% (95% CI = 79% to 91%)

Random effects (DerSimonian-Laird)

Pooled risk difference = 0.26218 (95% CI = 0.182357 to 0.342004)
Z (test Risk Difference) = 6.437507 $P < 0.0001$

Summary meta-analysis plot [random effects]

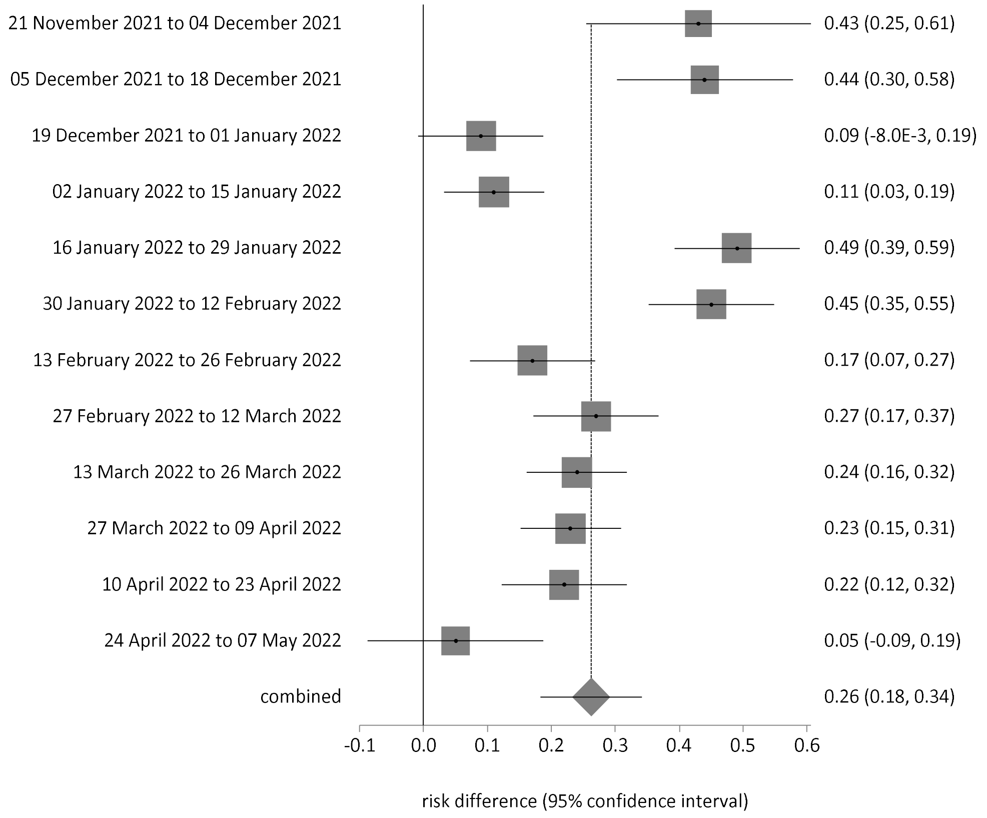

Multigenerational households v not

Time period	Risk Difference	SE	Approximate 95% CI		
1	0.02	0.07	-0.117197	0.157197	21 November 2021 to 04 December 2021
2	-0.09	0.06	-0.207598	0.027598	05 December 2021 to 18 December 2021
3	-0.14	0.05	-0.237998	-0.042002	19 December 2021 to 01 January 2022
4	-0.05	0.04	-0.128399	0.028399	02 January 2022 to 15 January 2022
5	-0.04	0.04	-0.118399	0.038399	16 January 2022 to 29 January 2022
6	0.06	0.04	-0.018399	0.138399	30 January 2022 to 12 February 2022
7	0.04	0.05	-0.057998	0.137998	13 February 2022 to 26 February 2022
8	-0.14	0.05	-0.237998	-0.042002	27 February 2022 to 12 March 2022
9	-0.12	0.04	-0.198399	-0.041601	13 March 2022 to 26 March 2022
10	-0.06	0.05	-0.157998	0.037998	27 March 2022 to 09 April 2022
11	-0.02	0.05	-0.117998	0.077998	10 April 2022 to 23 April 2022
12	-0.18	0.09	-0.356397	-0.003603	24 April 2022 to 07 May 2022

Non-combinability of studies

Cochran Q = 24.320219 (df = 11) P = 0.0114
Moment-based estimate of between studies variance = 0.002885
I_2 (inconsistency) = 54.8% (95% CI = 0% to 74.8%)

Random effects (DerSimonian-Laird)

Pooled risk difference = -0.054951 (95% CI = -0.096796 to -0.013106)
Z (test Risk Difference) = -2.573816 P = 0.0101

Summary meta-analysis plot [random effects]

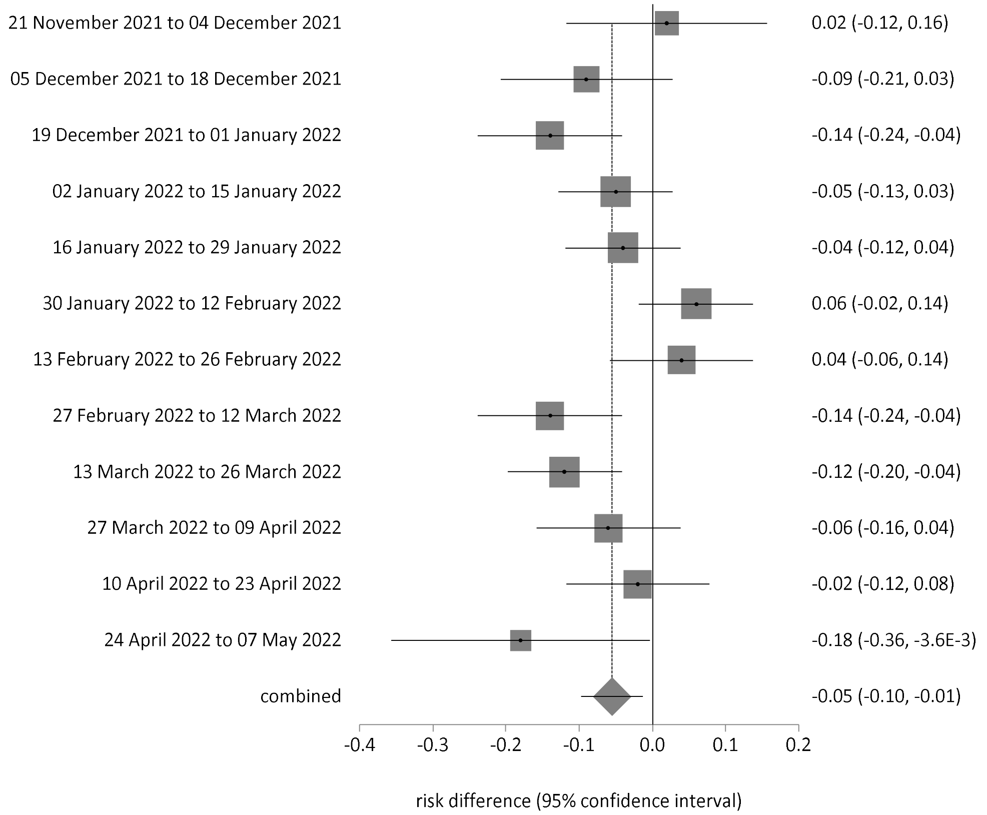

Ethnic minority v not

Time period	Risk Difference	SE	Approximate 95% CI		
1	-0.17	0.08	-0.326797	-0.013203	21 November 2021 to 04 December 2021
2	-0.29	0.06	-0.407598	-0.172402	05 December 2021 to 18 December 2021
3	-0.12	0.05	-0.217998	-0.022002	19 December 2021 to 01 January 2022
4	0.1	0.04	0.021601	0.178399	02 January 2022 to 15 January 2022
5	0.09	0.04	0.011601	0.168399	16 January 2022 to 29 January 2022
6	-0.1	0.04	-0.178399	-0.021601	30 January 2022 to 12 February 2022
7	-0.09	0.05	-0.187998	0.007998	13 February 2022 to 26 February 2022
8	-0.3	0.05	-0.397998	-0.202002	27 February 2022 to 12 March 2022
9	-0.27	0.04	-0.348399	-0.191601	13 March 2022 to 26 March 2022
10	-0.16	0.04	-0.238399	-0.081601	27 March 2022 to 09 April 2022
11	-0.14	0.05	-0.237998	-0.042002	10 April 2022 to 23 April 2022
12	0.01	0.07	-0.127197	0.147197	24 April 2022 to 07 May 2022

Non-combinability of studies

Cochran Q = 97.660268 (df = 11) $P < 0.0001$
Moment-based estimate of between studies variance = 0.017857
I_2 (inconsistency) = 88.7% (95% CI = 82.5% to 92%)

Random effects (DerSimonian-Laird)

Pooled risk difference = -0.118735 (95% CI = -0.199754 to -0.037716)
Z (test Risk Difference) = -2.872381 $P = 0.0041$

Summary meta-analysis plot [random effects]

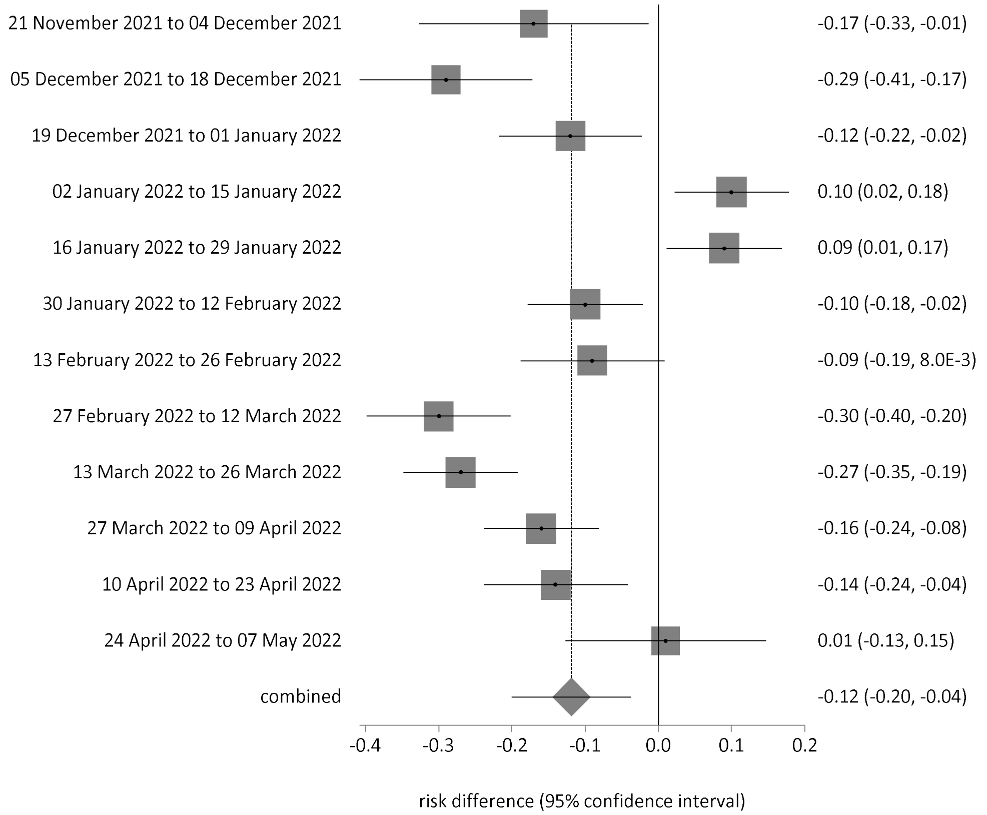

Employed, not working v Employed, working

Time period	Risk Difference	SE	Approximate 95% CI		
1	0.2	0.19	-0.172393	0.572393	21 November 2021 to 04 December 2021
2	-0.06	0.17	-0.393194	0.273194	05 December 2021 to 18 December 2021
3	-0.08	0.13	-0.334795	0.174795	19 December 2021 to 01 January 2022
4	0.15	0.09	-0.026397	0.326397	02 January 2022 to 15 January 2022
5	0.28	0.1	0.084004	0.475996	16 January 2022 to 29 January 2022
6	0.02	0.11	-0.195596	0.235596	30 January 2022 to 12 February 2022
7	0.02	0.13	-0.234795	0.274795	13 February 2022 to 26 February 2022
8	0.05	0.11	-0.165596	0.265596	27 February 2022 to 12 March 2022
9	0	0.1	-0.195996	0.195996	13 March 2022 to 26 March 2022
10	-0.07	0.1	-0.265996	0.125996	27 March 2022 to 09 April 2022
11	-0.18	0.13	-0.434795	0.074795	10 April 2022 to 23 April 2022
12	0.29	0.16	-0.023594	0.603594	24 April 2022 to 07 May 2022

Non-combinability of studies

Cochran Q = 15.757053 (df = 11) P = 0.1504

Moment-based estimate of between studies variance = 0.006071

I_2 (inconsistency) = 30.2% (95% CI = 0% to 63.9%)

Random effects (DerSimonian-Laird)

Pooled risk difference = 0.050259 (95% CI = -0.030999 to 0.131516)

Z (test Risk Difference) = 1.212266 P = 0.2254

Summary meta-analysis plot [random effects]

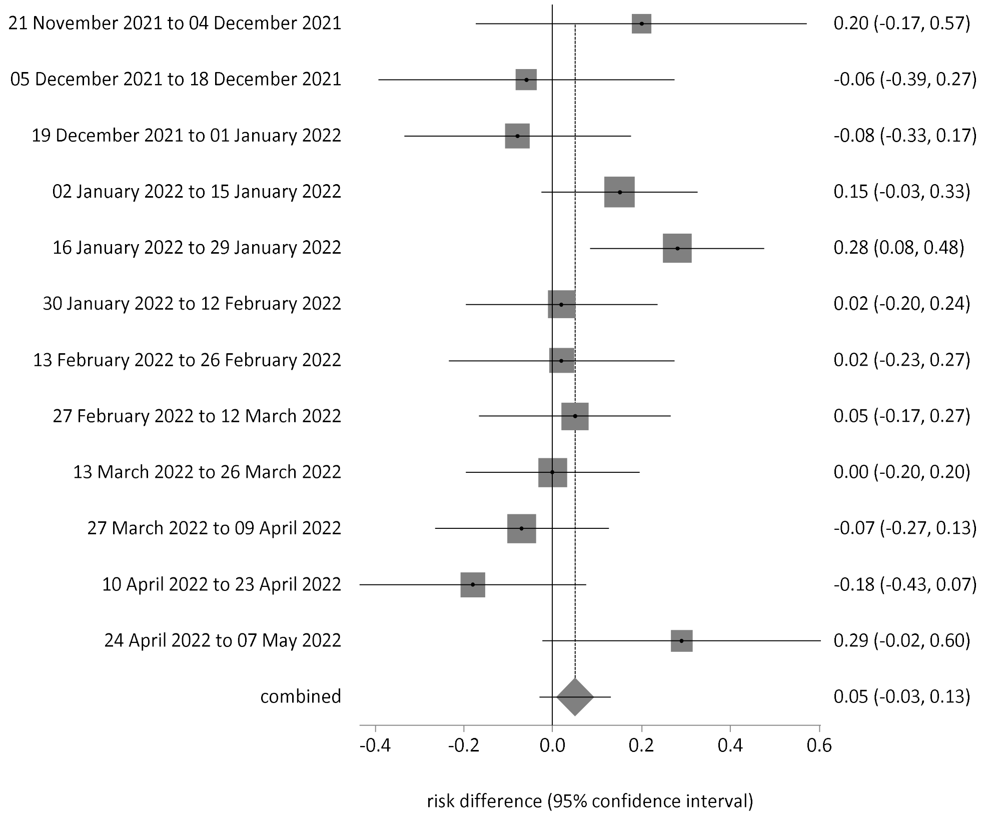

Not working v Employed, working

Time period	Risk Difference	SE	Approximate 95% CI		
1	-0.21	0.11	-0.425596	0.005596	21 November 2021 to 04 December 2021
2	-0.36	0.1	-0.555996	-0.164004	05 December 2021 to 18 December 2021
3	-0.47	0.07	-0.607197	-0.332803	19 December 2021 to 01 January 2022
4	-0.24	0.05	-0.337998	-0.142002	02 January 2022 to 15 January 2022
5	-0.07	0.06	-0.187598	0.047598	16 January 2022 to 29 January 2022
6	-0.08	0.06	-0.197598	0.037598	30 January 2022 to 12 February 2022
7	-0.26	0.07	-0.397197	-0.122803	13 February 2022 to 26 February 2022
8	-0.31	0.07	-0.447197	-0.172803	27 February 2022 to 12 March 2022
9	-0.2	0.05	-0.297998	-0.102002	13 March 2022 to 26 March 2022
10	-0.15	0.05	-0.247998	-0.052002	27 March 2022 to 09 April 2022
11	0	0.06	-0.117598	0.117598	10 April 2022 to 23 April 2022
12	-0.09	0.09	-0.266397	0.086397	24 April 2022 to 07 May 2022

Non-combinability of studies

Cochran Q = 43.151235 (df = 11) $P < 0.0001$
Moment-based estimate of between studies variance = 0.012099
I_2 (inconsistency) = 74.5% (95% CI = 49.9% to 84.2%)

Random effects (DerSimonian-Laird)

Pooled risk difference = -0.198226 (95% CI = -0.271825 to -0.124627)
Z (test Risk Difference) = -5.278814 $P < 0.0001$

Summary meta-analysis plot [random effects]

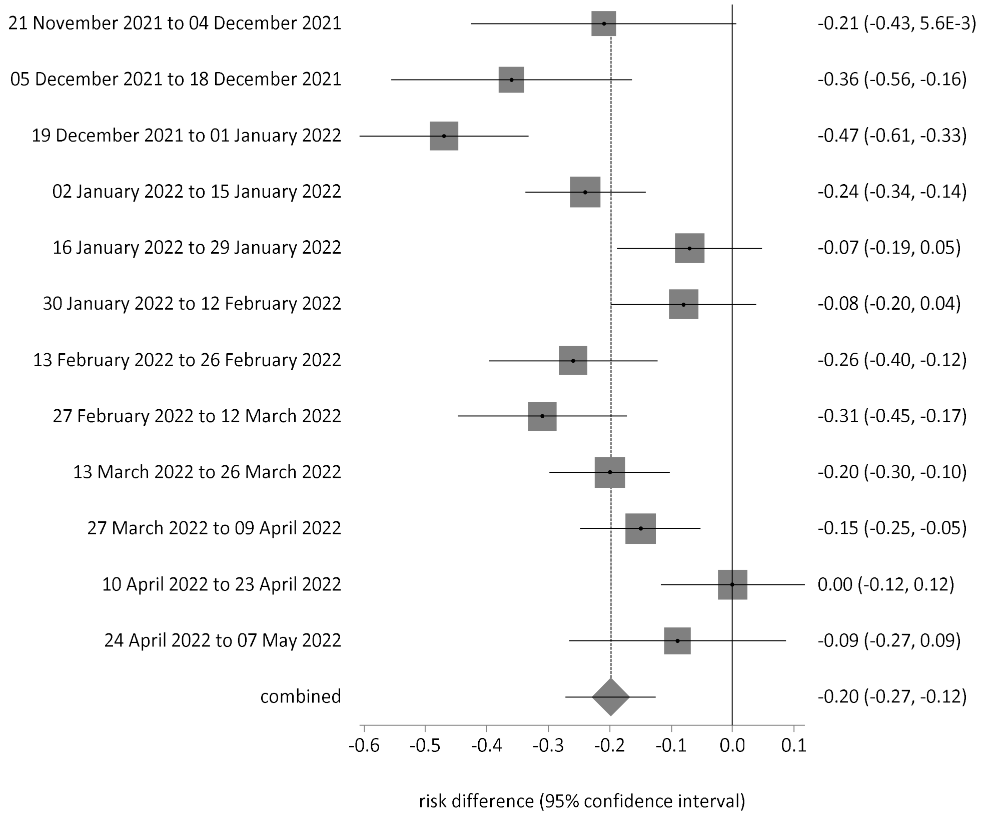

Child/student **v Employed, working**

Time period	Risk Difference	SE	Approximate 95% CI		
1	-0.21	0.2	-0.601993	0.181993	21 November 2021 to 04 December 2021
2	-0.13	0.16	-0.443594	0.183594	05 December 2021 to 18 December 2021
3	-0.4	0.08	-0.556797	-0.243203	19 December 2021 to 01 January 2022
4	-0.52	0.08	-0.676797	-0.363203	02 January 2022 to 15 January 2022
5	-0.18	0.11	-0.395596	0.035596	16 January 2022 to 29 January 2022
6	-0.12	0.1	-0.315996	0.075996	30 January 2022 to 12 February 2022
7	-0.09	0.08	-0.246797	0.066797	13 February 2022 to 26 February 2022
8	0.08	0.11	-0.135596	0.295596	27 February 2022 to 12 March 2022
9	-0.01	0.09	-0.186397	0.166397	13 March 2022 to 26 March 2022
10	-0.08	0.09	-0.256397	0.096397	27 March 2022 to 09 April 2022
11	-0.01	0.12	-0.245196	0.225196	10 April 2022 to 23 April 2022
12	-0.26	0.13	-0.514795	-0.005205	24 April 2022 to 07 May 2022

Non-combinability of studies

Cochran Q = 40.129609 (df = 11) $P < 0.0001$
Moment-based estimate of between studies variance = 0.027581
I_2 (inconsistency) = 72.6% (95% CI = 44.7% to 83.3%)

Random effects (DerSimonian-Laird)

Pooled risk difference = -0.164625 (95% CI = -0.277548 to -0.051702)
Z (test Risk Difference) = -2.857341 $P = 0.0043$

Summary meta-analysis plot [random effects]

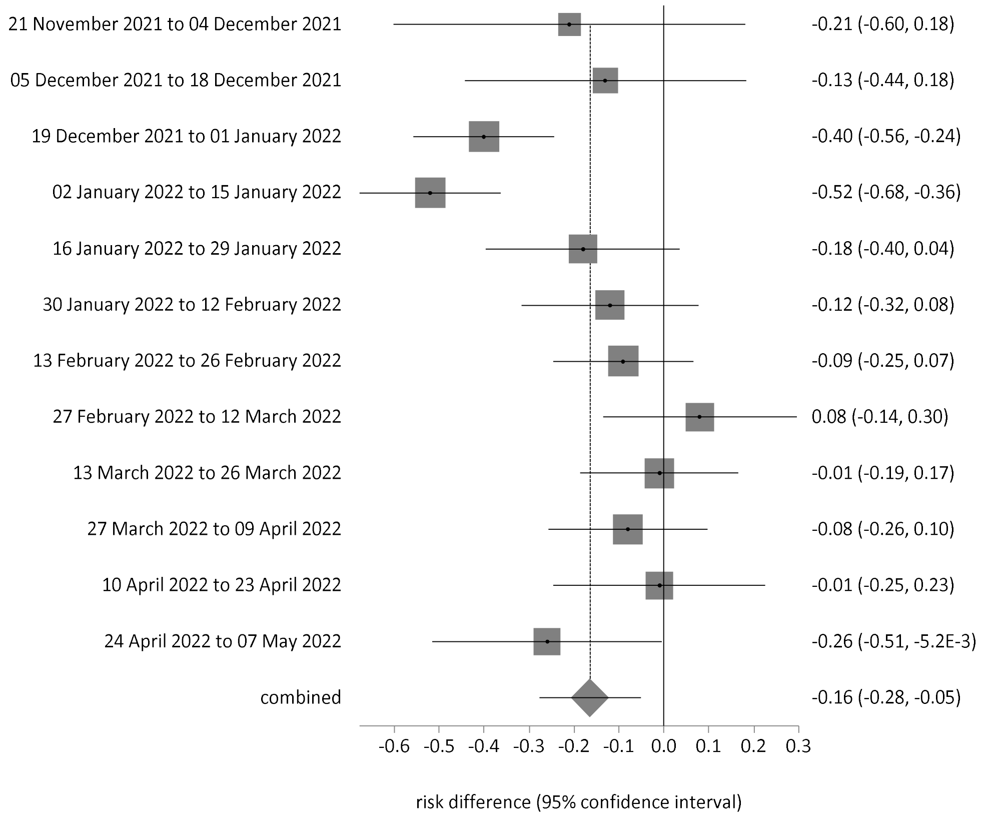

Rural town v major urban area

Time period	Risk Difference	SE	Approximate 95% CI		
1	0.15	0.08	-0.006797	0.306797	21 November 2021 to 04 December 2021
2	-0.15	0.08	-0.306797	0.006797	05 December 2021 to 18 December 2021
3	-0.21	0.06	-0.327598	-0.092402	19 December 2021 to 01 January 2022
4	-0.23	0.04	-0.308399	-0.151601	02 January 2022 to 15 January 2022
5	-0.03	0.05	-0.127998	0.067998	16 January 2022 to 29 January 2022
6	-0.14	0.05	-0.237998	-0.042002	30 January 2022 to 12 February 2022
7	-0.08	0.06	-0.197598	0.037598	13 February 2022 to 26 February 2022
8	-0.04	0.05	-0.137998	0.057998	27 February 2022 to 12 March 2022
9	0.14	0.04	0.061601	0.218399	13 March 2022 to 26 March 2022
10	0.06	0.04	-0.018399	0.138399	27 March 2022 to 09 April 2022
11	0.05	0.05	-0.047998	0.147998	10 April 2022 to 23 April 2022
12	0.08	0.07	-0.057197	0.217197	24 April 2022 to 07 May 2022

Non-combinability of studies

Cochran Q = 75.016785 (df = 11) $P < 0.0001$
Moment-based estimate of between studies variance = 0.015634
I_2 (inconsistency) = 85.3% (95% CI = 75.7% to 90%)

Random effects (DerSimonian-Laird)

Pooled risk difference = -0.034078 (95% CI = -0.111666 to 0.04351)
Z (test Risk Difference) = -0.86085 P = 0.3893

Summary meta-analysis plot [random effects]

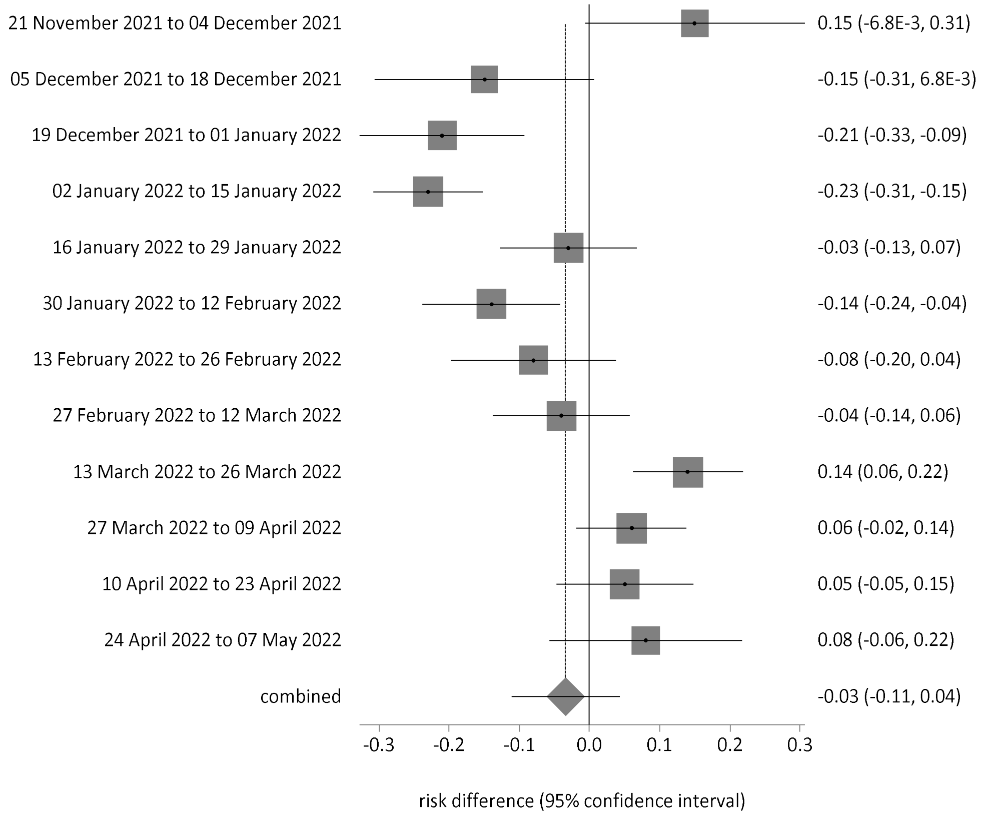

Rural town v major urban area

Time period	Risk Difference	SE	Approximate 95% CI		
1	0.16	0.08	0.003203	0.316797	21 November 2021 to 04 December 2021
2	-0.08	0.08	-0.236797	0.076797	05 December 2021 to 18 December 2021
3	-0.37	0.07	-0.507197	-0.232803	19 December 2021 to 01 January 2022
4	-0.36	0.04	-0.438399	-0.281601	02 January 2022 to 15 January 2022
5	-0.17	0.05	-0.267998	-0.072002	16 January 2022 to 29 January 2022
6	-0.15	0.05	-0.247998	-0.052002	30 January 2022 to 12 February 2022
7	-0.05	0.05	-0.147998	0.047998	13 February 2022 to 26 February 2022
8	-0.03	0.05	-0.127998	0.067998	27 February 2022 to 12 March 2022
9	0.08	0.04	0.001601	0.158399	13 March 2022 to 26 March 2022
10	-0.02	0.04	-0.098399	0.058399	27 March 2022 to 09 April 2022
11	0.02	0.05	-0.077998	0.117998	10 April 2022 to 23 April 2022
12	0.05	0.07	-0.087197	0.187197	24 April 2022 to 07 May 2022

Non-combinability of studies

Cochran Q = 107.289996 (df = 11) $P < 0.0001$
Moment-based estimate of between studies variance = 0.023271
I_2 (inconsistency) = 89.7% (95% CI = 84.4% to 92.6%)

Random effects (DerSimonian-Laird)

Pooled risk difference = -0.078294 (95% CI = -0.170377 to 0.013788)
Z (test Risk Difference) = -1.666488 $P = 0.0956$

Summary meta-analysis plot [random effects]

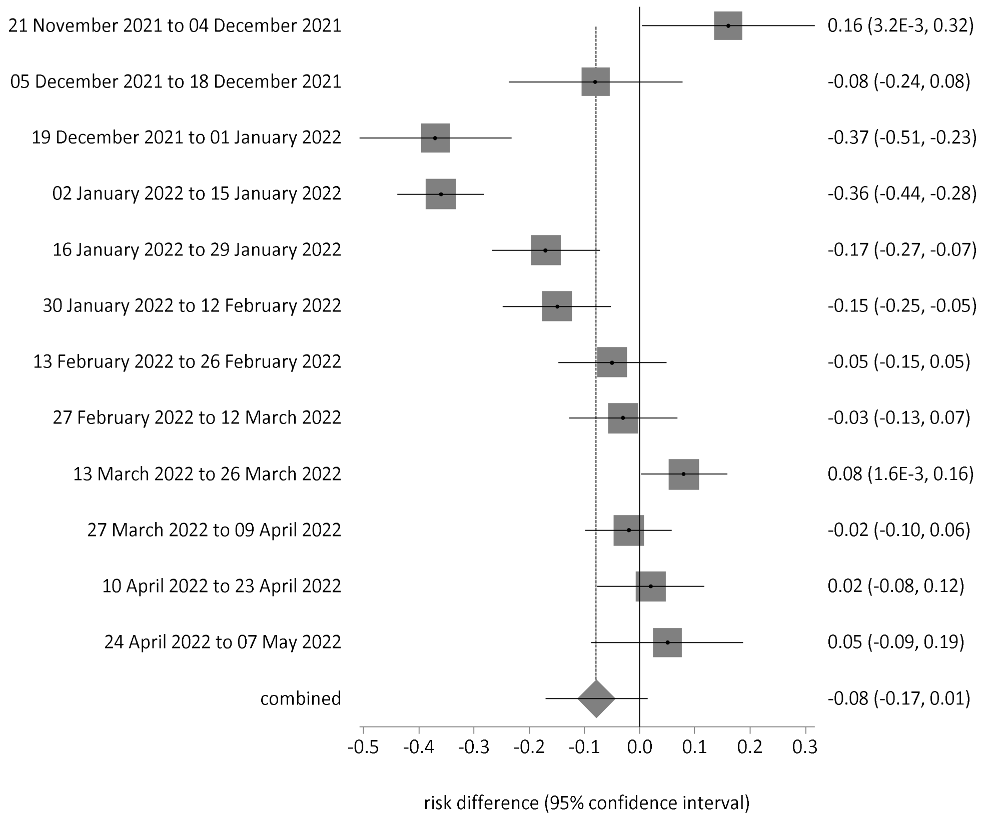

Rural village v major urban area

Time period	Risk Difference	SE	Approximate 95% CI		
1	0.16	0.08	0.003203	0.316797	21 November 2021 to 04 December 2021
2	-0.08	0.08	-0.236797	0.076797	05 December 2021 to 18 December 2021
3	-0.37	0.07	-0.507197	-0.232803	19 December 2021 to 01 January 2022
4	-0.36	0.04	-0.438399	-0.281601	02 January 2022 to 15 January 2022
5	-0.17	0.05	-0.267998	-0.072002	16 January 2022 to 29 January 2022
6	-0.15	0.05	-0.247998	-0.052002	30 January 2022 to 12 February 2022
7	-0.05	0.05	-0.147998	0.047998	13 February 2022 to 26 February 2022
8	-0.03	0.05	-0.127998	0.067998	27 February 2022 to 12 March 2022
9	0.08	0.04	0.001601	0.158399	13 March 2022 to 26 March 2022
10	-0.02	0.04	-0.098399	0.058399	27 March 2022 to 09 April 2022
11	0.02	0.05	-0.077998	0.117998	10 April 2022 to 23 April 2022
12	0.05	0.07	-0.087197	0.187197	24 April 2022 to 07 May 2022

Non-combinability of studies

Cochran Q = 107.289996 (df = 11) $P < 0.0001$
Moment-based estimate of between studies variance = 0.023271
I_2 (inconsistency) = 89.7% (95% CI = 84.4% to 92.6%)

Random effects (DerSimonian-Laird)

Pooled risk difference = -0.078294 (95% CI = -0.170377 to 0.013788)
Z (test Risk Difference) = -1.666488 $P = 0.0956$

Summary meta-analysis plot [random effects]

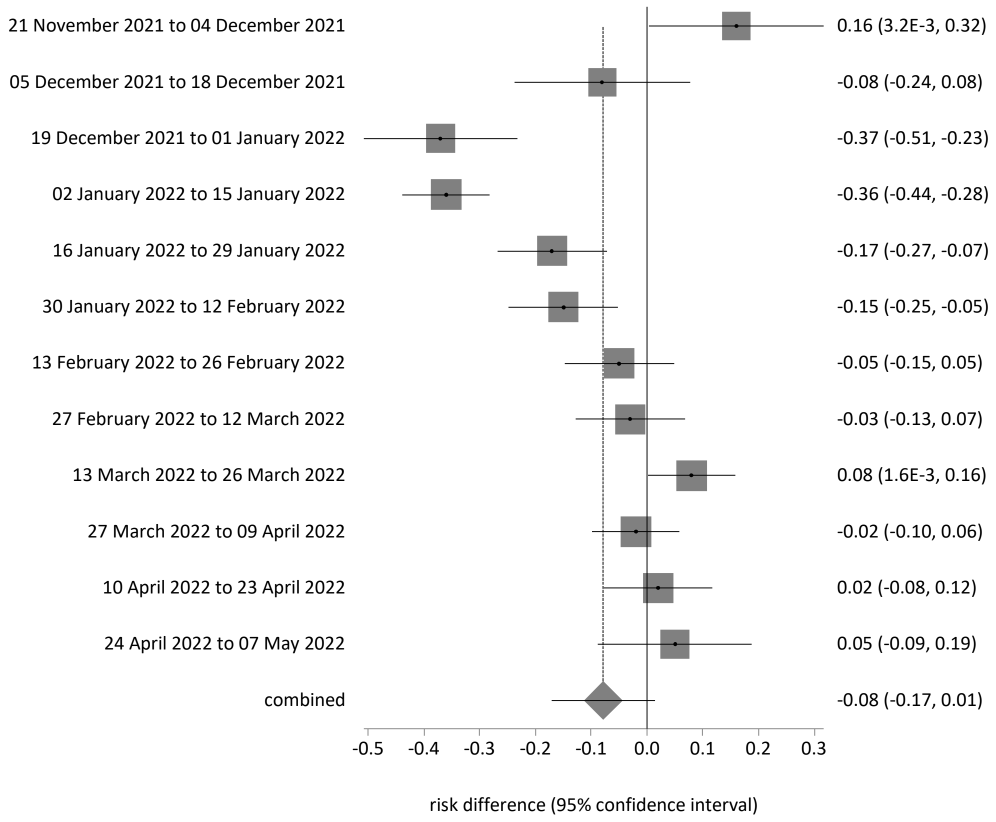

Work in a health or social care role

Time period	Risk Difference	SE	Approximate 95% CI		
1	-0.54	0.13	-0.794795	-0.285205	21 November 2021 to 04 December 2021
2	-0.39	0.1	-0.585996	-0.194004	05 December 2021 to 18 December 2021
3	-0.06	0.07	-0.197197	0.077197	19 December 2021 to 01 January 2022
4	0.18	0.05	0.082002	0.277998	02 January 2022 to 15 January 2022
5	0.03	0.06	-0.087598	0.147598	16 January 2022 to 29 January 2022
6	0.03	0.06	-0.087598	0.147598	30 January 2022 to 12 February 2022
7	0.16	0.06	0.042402	0.277598	13 February 2022 to 26 February 2022
8	0.1	0.06	-0.017598	0.217598	27 February 2022 to 12 March 2022
9	0.07	0.05	-0.027998	0.167998	13 March 2022 to 26 March 2022
10	0.05	0.05	-0.047998	0.147998	27 March 2022 to 09 April 2022
11	-0.04	0.06	-0.157598	0.077598	10 April 2022 to 23 April 2022
12	0.05	0.08	-0.106797	0.206797	24 April 2022 to 07 May 2022

Non-combinability of studies

Cochran Q = 55.523886 (df = 11) $P < 0.0001$
Moment-based estimate of between studies variance = 0.015877
I_2 (inconsistency) = 80.2% (95% CI = 64.1% to 87.2%)

Random effects (DerSimonian-Laird)

Pooled risk difference = -0.002558 (95% CI = -0.083792 to 0.078676)
Z (test Risk Difference) = -0.061716 $P = 0.9508$

Summary meta-analysis plot [random effects]

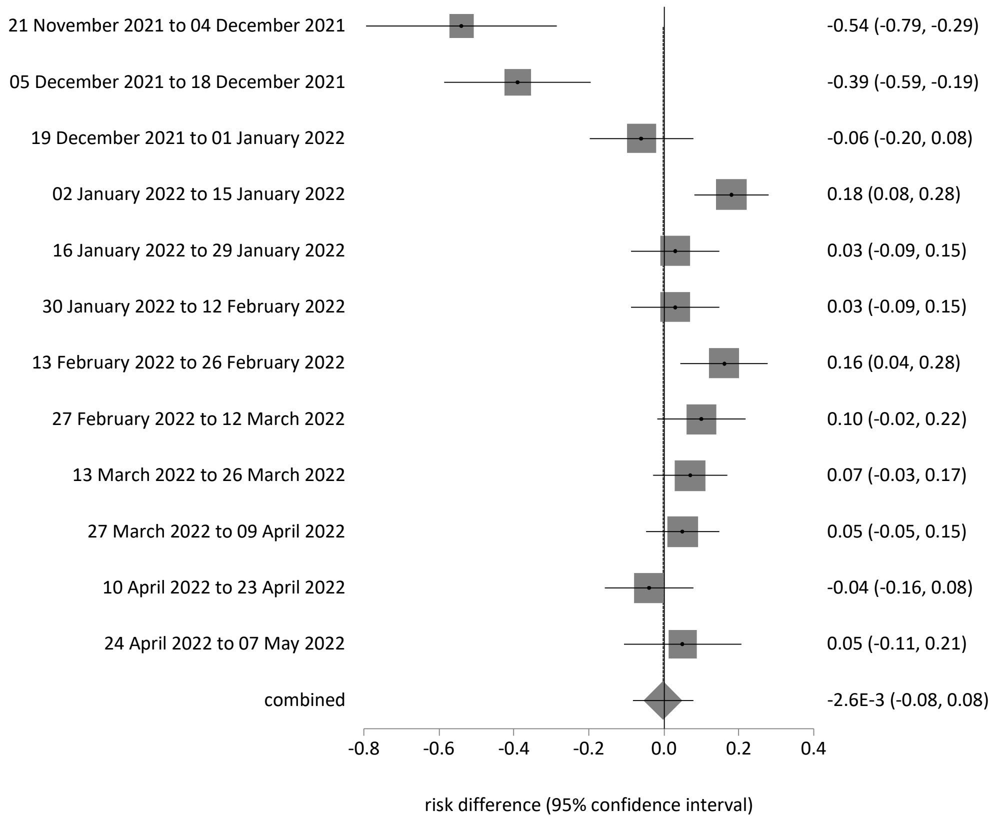

Work in a patient-facing role

Time period	Risk Difference	SE	Approximate 95% CI		
1	-0.46	0.16	-0.773594	-0.146406	21 November 2021 to 04 December 2021
2	-0.28	0.13	-0.534795	-0.025205	05 December 2021 to 18 December 2021
3	-0.11	0.09	-0.286397	0.066397	19 December 2021 to 01 January 2022
4	0.13	0.06	0.012402	0.247598	02 January 2022 to 15 January 2022
5	0.03	0.08	-0.126797	0.186797	16 January 2022 to 29 January 2022
6	0.12	0.07	-0.017197	0.257197	30 January 2022 to 12 February 2022
7	0.28	0.08	0.123203	0.436797	13 February 2022 to 26 February 2022
8	0.12	0.08	-0.036797	0.276797	27 February 2022 to 12 March 2022
9	0.12	0.06	0.002402	0.237598	13 March 2022 to 26 March 2022
10	0.13	0.06	0.012402	0.247598	27 March 2022 to 09 April 2022
11	-0.03	0.08	-0.186797	0.126797	10 April 2022 to 23 April 2022
12	-0.02	0.11	-0.235596	0.195596	24 April 2022 to 07 May 2022

Non-combinability of studies

Cochran Q = 35.175757 (df = 11) P = 0.0002
Moment-based estimate of between studies variance = 0.013702
I_2 (inconsistency) = 68.7% (95% CI = 33.9% to 81.4%)

Random effects (DerSimonian-Laird)

Pooled risk difference = 0.038036 (95% CI = -0.044138 to 0.12021)
Z (test Risk Difference) = 0.907215 P = 0.3643

Summary meta-analysis plot [random effects]

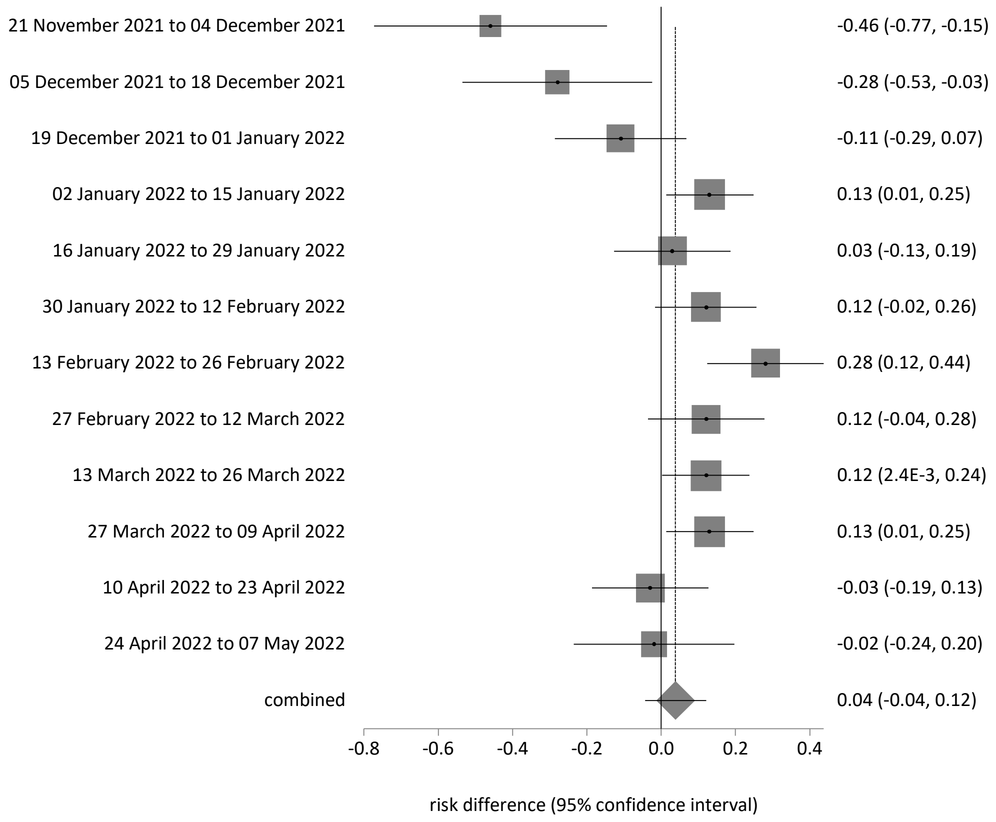

Work in a care or nursing home

Time period	Risk Difference	SE	Approximate 95% CI		
1	-0.99	0.36	-1.695587	-0.284413	21 November 2021 to 04 December 2021
2	-0.49	0.23	-0.940792	-0.039208	05 December 2021 to 18 December 2021
3	-0.19	0.16	-0.503594	0.123594	19 December 2021 to 01 January 2022
4	0.13	0.1	-0.065996	0.325996	02 January 2022 to 15 January 2022
5	0.13	0.12	-0.105196	0.365196	16 January 2022 to 29 January 2022
6	0.07	0.12	-0.165196	0.305196	30 January 2022 to 12 February 2022
7	0.23	0.12	-0.005196	0.465196	13 February 2022 to 26 February 2022
8	0.24	0.11	0.024404	0.455596	27 February 2022 to 12 March 2022
9	-0.04	0.11	-0.255596	0.175596	13 March 2022 to 26 March 2022
10	0.02	0.1	-0.175996	0.215996	27 March 2022 to 09 April 2022
11	0.17	0.12	-0.065196	0.405196	10 April 2022 to 23 April 2022
12	-0.05	0.18	-0.402794	0.302794	24 April 2022 to 07 May 2022

Non-combinability of studies

Cochran Q = 24.376938 (df = 11) P = 0.0112
Moment-based estimate of between studies variance = 0.020143
I_2 (inconsistency) = 54.9% (95% CI = 0% to 74.9%)

Random effects (DerSimonian-Laird)

Pooled risk difference = 0.033146 (95% CI = -0.079001 to 0.145292)
Z (test Risk Difference) = 0.579282 P = 0.5624

Summary meta-analysis plot [random effects]

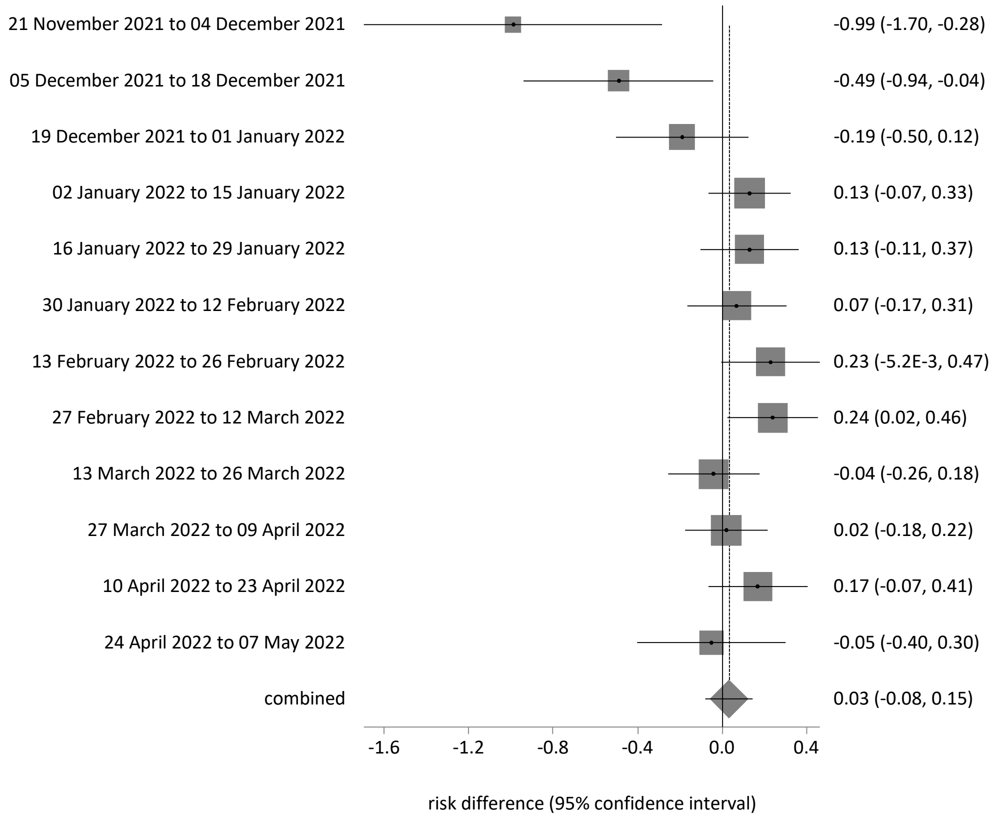

Work in direct contact with others

Time period	Risk Difference	SE	Approximate 95% CI		
1	0.02	0.06	-0.097598	0.137598	21 November 2021 to 04 December 2021
2	-0.09	0.05	-0.187998	0.007998	05 December 2021 to 18 December 2021
3	0.05	0.04	-0.028399	0.128399	19 December 2021 to 01 January 2022
4	0.17	0.03	0.111201	0.228799	02 January 2022 to 15 January 2022
5	0.2	0.04	0.121601	0.278399	16 January 2022 to 29 January 2022
6	0.11	0.03	0.051201	0.168799	30 January 2022 to 12 February 2022
7	0.09	0.04	0.011601	0.168399	13 February 2022 to 26 February 2022
8	0.02	0.03	-0.038799	0.078799	27 February 2022 to 12 March 2022
9	0.08	0.03	0.021201	0.138799	13 March 2022 to 26 March 2022
10	0.04	0.03	-0.018799	0.098799	27 March 2022 to 09 April 2022
11	-0.08	0.04	-0.158399	-0.001601	10 April 2022 to 23 April 2022
12	-0.03	0.05	-0.127998	0.067998	24 April 2022 to 07 May 2022

Non-combinability of studies

Cochran Q = 56.534164 (df = 11) P < 0.0001
Moment-based estimate of between studies variance = 0.005524
I_2 (inconsistency) = 80.5% (95% CI = 65% to 87.4%)

Random effects (DerSimonian-Laird)

Pooled risk difference = 0.052461 (95% CI = 0.004906 to 0.100015)
Z (test Risk Difference) = 2.162151 P = 0.0306

Summary meta-analysis plot [random effects]

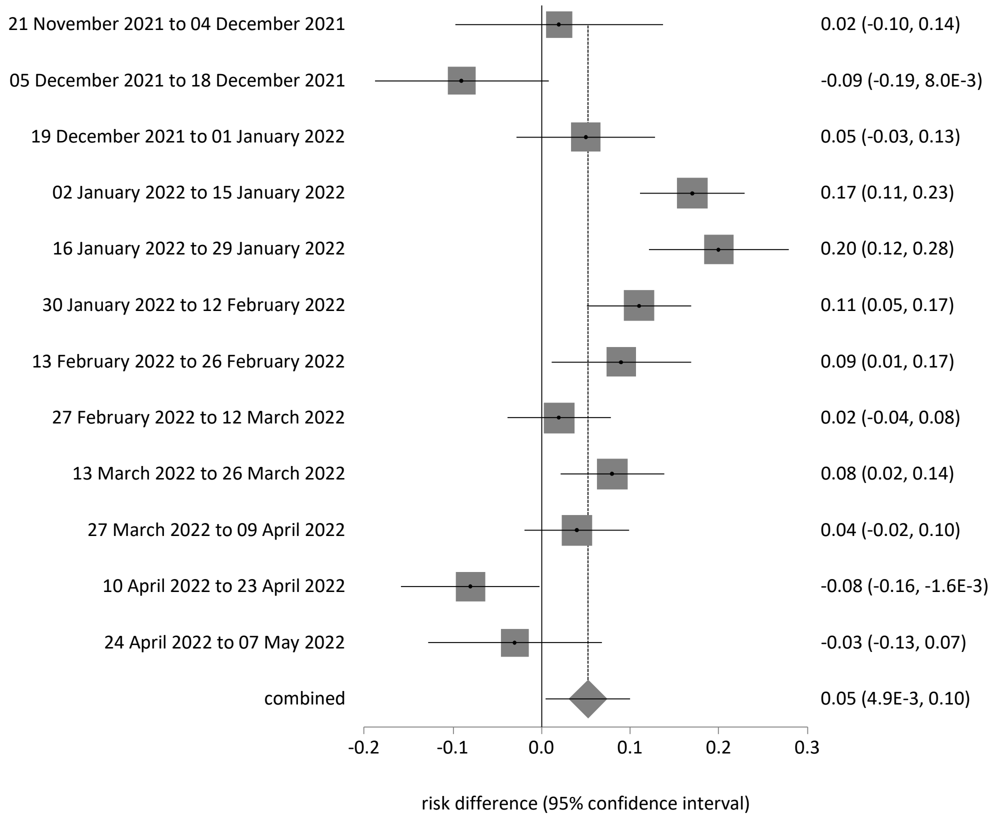

Contact with care homes in the last 28 days No I haven't, but someone else in my household has v No

Time period	Risk Difference	SE	Approximate 95% CI		
1	-0.24	0.14	-0.514395	0.034395	21 November 2021 to 04 December 2021
2	-0.06	0.11	-0.275596	0.155596	05 December 2021 to 18 December 2021
3	-0.3	0.11	-0.515596	-0.084404	19 December 2021 to 01 January 2022
4	0.05	0.07	-0.087197	0.187197	02 January 2022 to 15 January 2022
5	-0.07	0.08	-0.226797	0.086797	16 January 2022 to 29 January 2022
6	0.03	0.07	-0.107197	0.167197	30 January 2022 to 12 February 2022
7	-0.12	0.09	-0.296397	0.056397	13 February 2022 to 26 February 2022
8	0.02	0.08	-0.136797	0.176797	27 February 2022 to 12 March 2022
9	0.01	0.06	-0.107598	0.127598	13 March 2022 to 26 March 2022
10	-0.06	0.07	-0.197197	0.077197	27 March 2022 to 09 April 2022
11	-0.19	0.09	-0.366397	-0.013603	10 April 2022 to 23 April 2022
12	0.12	0.11	-0.095596	0.335596	24 April 2022 to 07 May 2022

Non-combinability of studies

Cochran Q = 17.475447 (df = 11) P = 0.0946
Moment-based estimate of between studies variance = 0.004113
I_2 (inconsistency) = 37.1% (95% CI = 0% to 66.9%)

Random effects (DerSimonian-Laird)

Pooled risk difference = -0.049389 (95% CI = -0.110058 to 0.01128)
Z (test Risk Difference) = -1.595554 P = 0.1106

Summary meta-analysis plot [random effects]

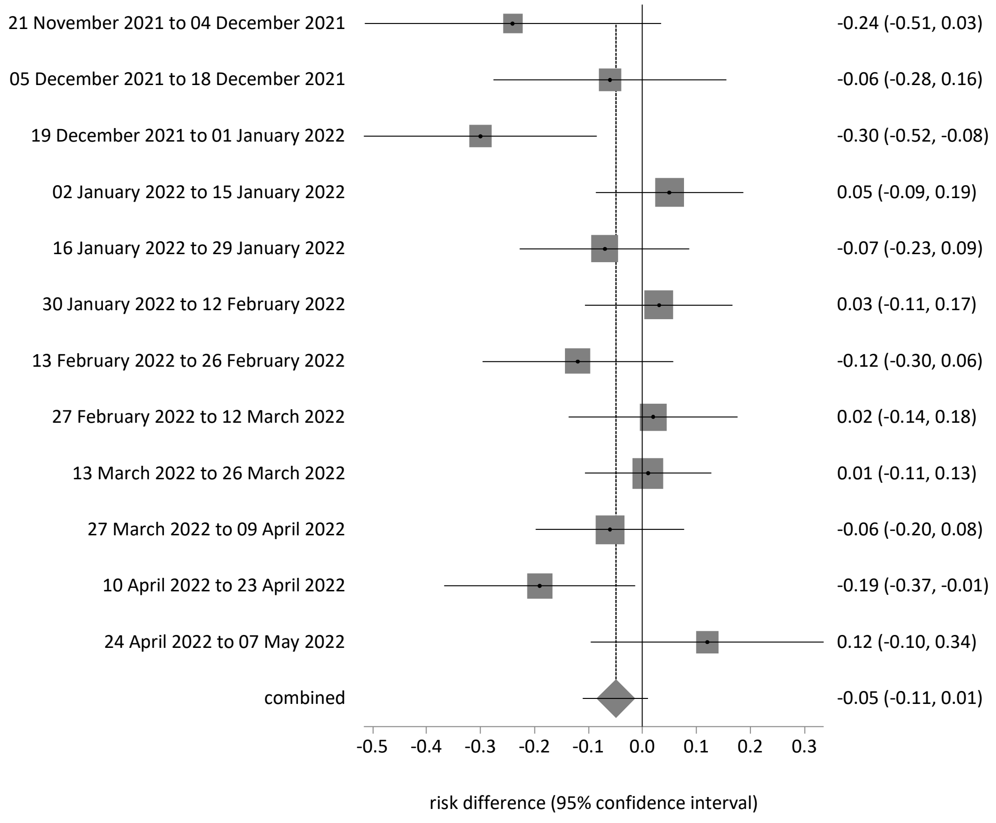

Contact with care homes in the last 28 days Yes, I have v No

Time period	Risk Difference	SE	Approximate 95% CI		
1	-0.55	0.16	-0.863594	-0.236406	21 November 2021 to 04 December 2021
2	-0.54	0.14	-0.814395	-0.265605	05 December 2021 to 18 December 2021
3	-0.11	0.08	-0.266797	0.046797	19 December 2021 to 01 January 2022
4	-0.03	0.06	-0.147598	0.087598	02 January 2022 to 15 January 2022
5	-0.11	0.08	-0.266797	0.046797	16 January 2022 to 29 January 2022
6	-0.22	0.08	-0.376797	-0.063203	30 January 2022 to 12 February 2022
7	-0.05	0.08	-0.206797	0.106797	13 February 2022 to 26 February 2022
8	-0.05	0.07	-0.187197	0.087197	27 February 2022 to 12 March 2022
9	-0.11	0.06	-0.227598	0.007598	13 March 2022 to 26 March 2022
10	-0.17	0.06	-0.287598	-0.052402	27 March 2022 to 09 April 2022
11	-0.11	0.07	-0.247197	0.027197	10 April 2022 to 23 April 2022
12	0.02	0.08	-0.136797	0.176797	24 April 2022 to 07 May 2022

Non-combinability of studies

Cochran Q = 25.565527 (df = 11) P = 0.0075
Moment-based estimate of between studies variance = 0.00765
I_2 (inconsistency) = 57% (95% CI = 0% to 75.8%)

Random effects (DerSimonian-Laird)

Pooled risk difference = -0.129748 (95% CI = -0.197026 to -0.062471)
Z (test Risk Difference) = -3.779893 P = 0.0002

Summary meta-analysis plot [random effects]

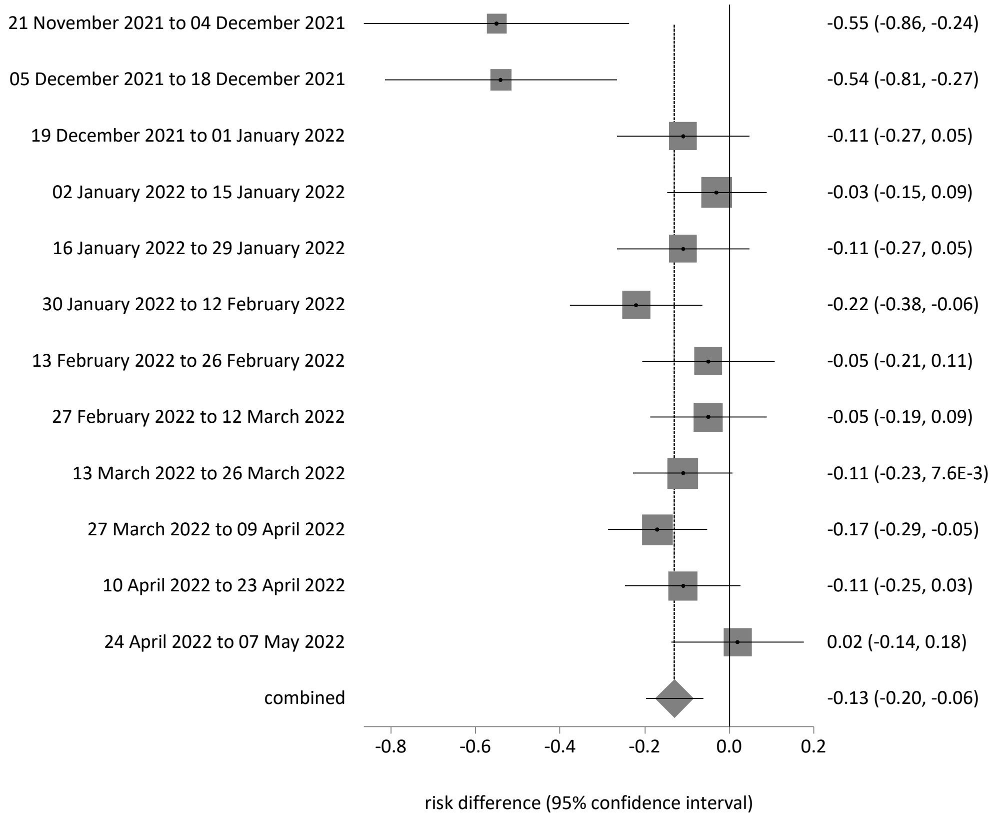

Contact with hospitals in the last 28 days No I haven't, but someone else in my household has v No

Time period	Risk Difference	SE	Approximate 95% CI		
1	-0.17	0.07	-0.307197	-0.032803	21 November 2021 to 04 December 2021
2	0.02	0.06	-0.097598	0.137598	05 December 2021 to 18 December 2021
3	-0.17	0.05	-0.267998	-0.072002	19 December 2021 to 01 January 2022
4	-0.09	0.04	-0.168399	-0.011601	02 January 2022 to 15 January 2022
5	-0.15	0.04	-0.228399	-0.071601	16 January 2022 to 29 January 2022
6	-0.05	0.04	-0.128399	0.028399	30 January 2022 to 12 February 2022
7	-0.12	0.05	-0.217998	-0.022002	13 February 2022 to 26 February 2022
8	-0.12	0.04	-0.198399	-0.041601	27 February 2022 to 12 March 2022
9	-0.05	0.03	-0.108799	0.008799	13 March 2022 to 26 March 2022
10	-0.13	0.04	-0.208399	-0.051601	27 March 2022 to 09 April 2022
11	-0.1	0.04	-0.178399	-0.021601	10 April 2022 to 23 April 2022
12	-0.12	0.07	-0.257197	0.017197	24 April 2022 to 07 May 2022

Non-combinability of studies

Cochran Q = 13.965127 (df = 11) P = 0.2349
Moment-based estimate of between studies variance = 0.00052
I_2 (inconsistency) = 21.2% (95% CI = 0% to 59.9%)

Random effects (DerSimonian-Laird)

Pooled risk difference = -0.100194 (95% CI = -0.128456 to -0.071932)
Z (test Risk Difference) = -6.948467 P < 0.0001

Summary meta-analysis plot [random effects]

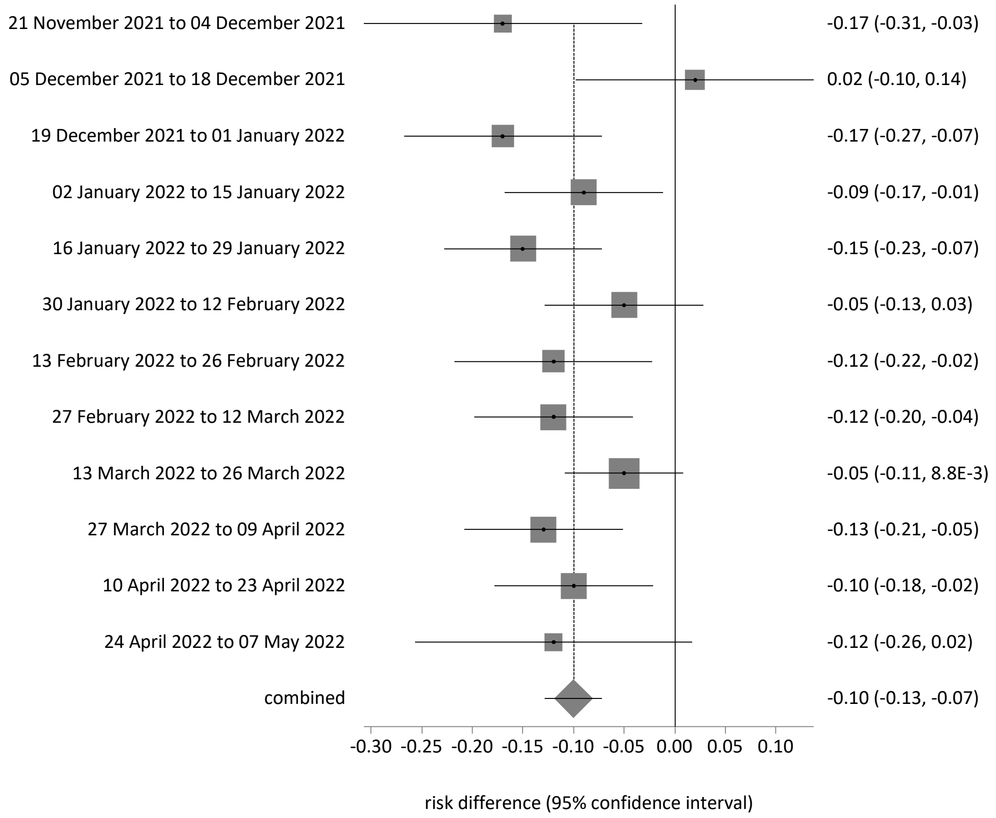

Contact with hospitals in the last 28 days Yes v No

Time period	Risk Difference	SE	Approximate 95% CI		
1	-0.28	0.06	-0.397598	-0.162402	21 November 2021 to 04 December 2021
2	-0.23	0.06	-0.347598	-0.112402	05 December 2021 to 18 December 2021
3	-0.22	0.04	-0.298399	-0.141601	19 December 2021 to 01 January 2022
4	-0.12	0.03	-0.178799	-0.061201	02 January 2022 to 15 January 2022
5	-0.19	0.04	-0.268399	-0.111601	16 January 2022 to 29 January 2022
6	-0.22	0.03	-0.278799	-0.161201	30 January 2022 to 12 February 2022
7	-0.1	0.04	-0.178399	-0.021601	13 February 2022 to 26 February 2022
8	-0.15	0.03	-0.208799	-0.091201	27 February 2022 to 12 March 2022
9	-0.17	0.03	-0.228799	-0.111201	13 March 2022 to 26 March 2022
10	-0.21	0.03	-0.268799	-0.151201	27 March 2022 to 09 April 2022
11	-0.16	0.03	-0.218799	-0.101201	10 April 2022 to 23 April 2022
12	-0.14	0.05	-0.237998	-0.042002	24 April 2022 to 07 May 2022

Non-combinability of studies

Cochran Q = 17.258957 (df = 11) P = 0.1004
Moment-based estimate of between studies variance = 0.000731
I_2 (inconsistency) = 36.3% (95% CI = 0% to 66.6%)

Random effects (DerSimonian-Laird)

Pooled risk difference = -0.176292 (95% CI = -0.20217 to -0.150415)
Z (test Risk Difference) = -13.352504 P < 0.0001

Summary meta-analysis plot [random effects]

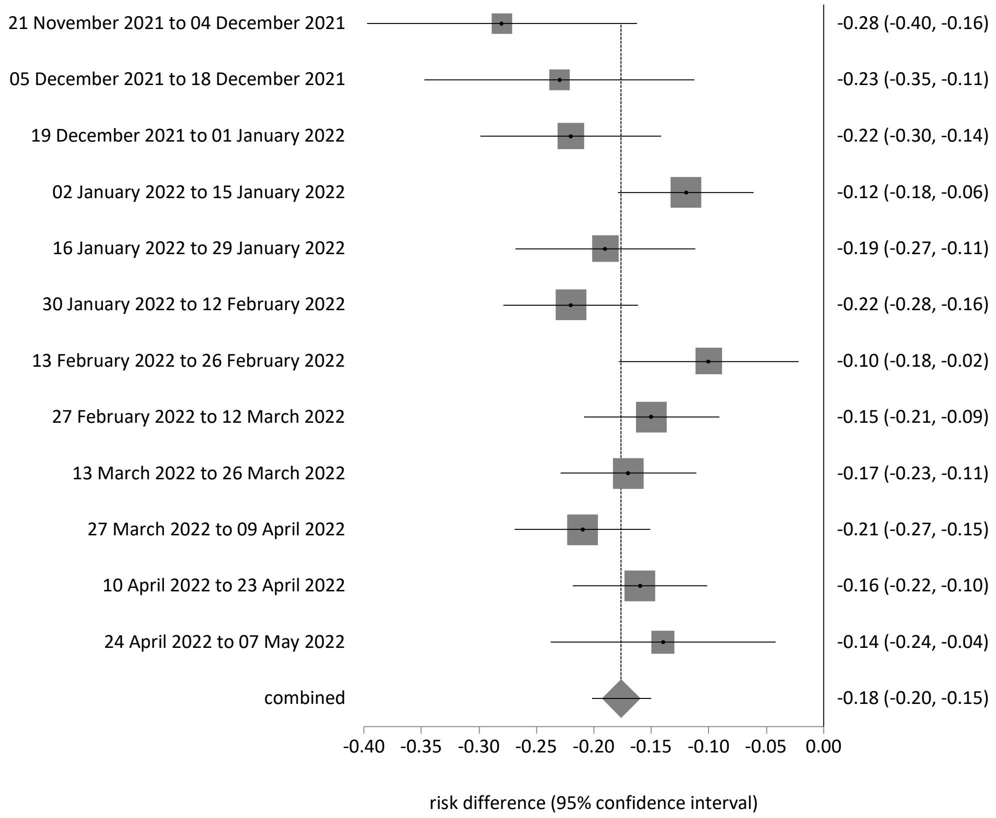

Smoking Status - only Vape v None

Time period	Risk Difference	SE	Approximate 95% CI		
1	-0.14	0.16	-0.453594	0.173594	21 November 2021 to 04 December 2021
2	-0.16	0.14	-0.434395	0.114395	05 December 2021 to 18 December 2021
3	0.03	0.09	-0.146397	0.206397	19 December 2021 to 01 January 2022
4	0.14	0.07	0.002803	0.277197	02 January 2022 to 15 January 2022
5	0.09	0.08	-0.066797	0.246797	16 January 2022 to 29 January 2022
6	-0.11	0.08	-0.266797	0.046797	30 January 2022 to 12 February 2022
7	-0.03	0.09	-0.206397	0.146397	13 February 2022 to 26 February 2022
8	-0.35	0.1	-0.545996	-0.154004	27 February 2022 to 12 March 2022
9	-0.12	0.07	-0.257197	0.017197	13 March 2022 to 26 March 2022
10	-0.21	0.07	-0.347197	-0.072803	27 March 2022 to 09 April 2022
11	-0.07	0.08	-0.226797	0.086797	10 April 2022 to 23 April 2022
12	-0.09	0.11	-0.305596	0.125596	24 April 2022 to 07 May 2022

Non-combinability of studies

Cochran Q = 27.663886 (df = 11) P = 0.0036
Moment-based estimate of between studies variance = 0.011535
I_2 (inconsistency) = 60.2% (95% CI = 8.3% to 77.4%)

Random effects (DerSimonian-Laird)

Pooled risk difference = -0.075982 (95% CI = -0.156033 to 0.004068)
Z (test Risk Difference) = -1.860351 P = 0.0628

Summary meta-analysis plot [random effects]

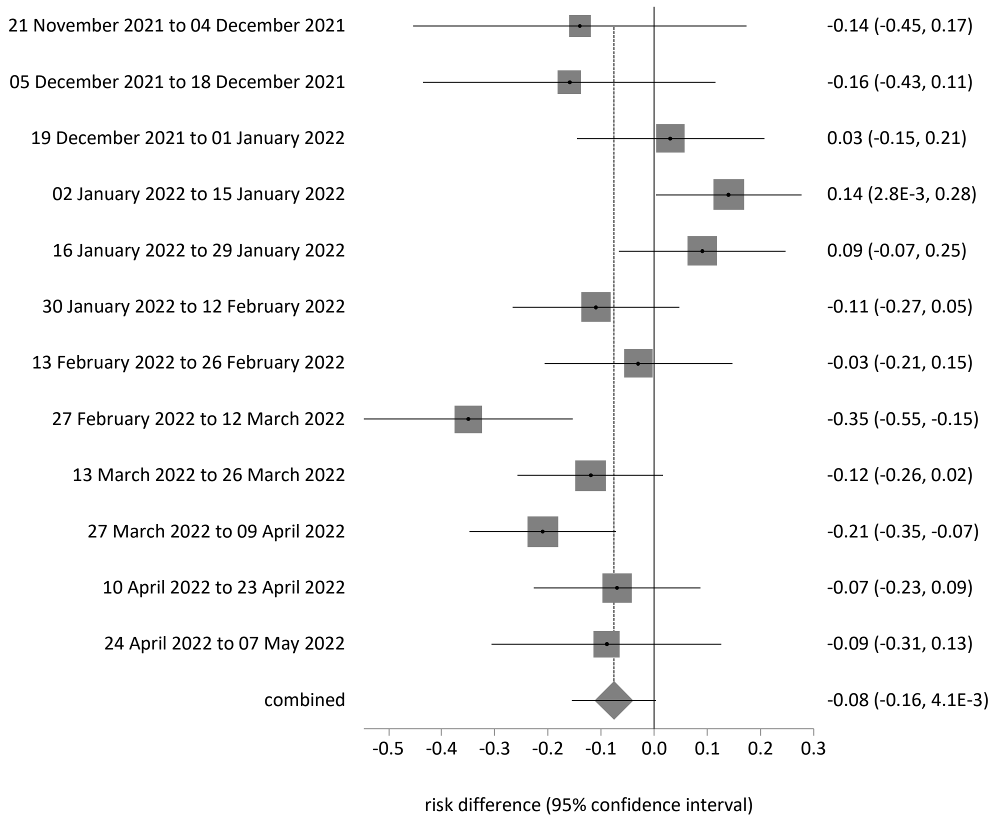

Smoking status tobacco smoker v none

Time period	Risk Difference	SE	Approximate 95% CI		
1	-0.37	0.11	-0.585596	-0.154404	21 November 2021 to 04 December 2021
2	-0.17	0.09	-0.346397	0.006397	05 December 2021 to 18 December 2021
3	-0.09	0.06	-0.207598	0.027598	19 December 2021 to 01 January 2022
4	0	0.04	-0.078399	0.078399	02 January 2022 to 15 January 2022
5	-0.06	0.06	-0.177598	0.057598	16 January 2022 to 29 January 2022
6	-0.11	0.05	-0.207998	-0.012002	30 January 2022 to 12 February 2022
7	-0.06	0.06	-0.177598	0.057598	13 February 2022 to 26 February 2022
8	-0.23	0.06	-0.347598	-0.112402	27 February 2022 to 12 March 2022
9	-0.22	0.05	-0.317998	-0.122002	13 March 2022 to 26 March 2022
10	-0.24	0.05	-0.337998	-0.142002	27 March 2022 to 09 April 2022
11	-0.28	0.06	-0.397598	-0.162402	10 April 2022 to 23 April 2022
12	-0.35	0.08	-0.506797	-0.193203	24 April 2022 to 07 May 2022

Non-combinability of studies

Cochran Q = 42.172773 (df = 11) $P < 0.0001$
Moment-based estimate of between studies variance = 0.009705
I_2 (inconsistency) = 73.9% (95% CI = 48.3% to 83.9%)

Random effects (DerSimonian-Laird)

Pooled risk difference = -0.170522 (95% CI = -0.236814 to -0.104231)
Z (test Risk Difference) = -5.041673 $P < 0.0001$

Summary meta-analysis plot [random effects]

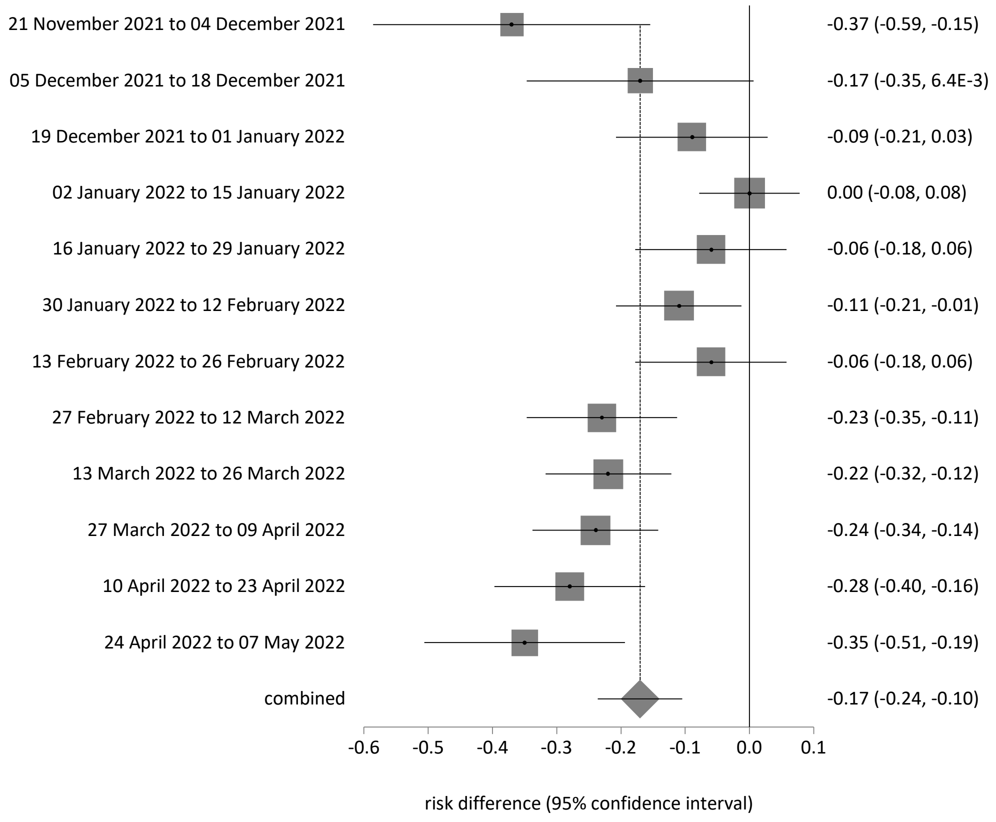

Disabled v not

Time period	Risk Difference	SE	Approximate 95% CI		
1	-0.13	0.08	-0.286797	0.026797	21 November 2021 to 04 December 2021
2	-0.27	0.08	-0.426797	-0.113203	05 December 2021 to 18 December 2021
3	-0.15	0.06	-0.267598	-0.032402	19 December 2021 to 01 January 2022
4	-0.08	0.04	-0.158399	-0.001601	02 January 2022 to 15 January 2022
5	-0.03	0.04	-0.108399	0.048399	16 January 2022 to 29 January 2022
6	-0.11	0.04	-0.188399	-0.031601	30 January 2022 to 12 February 2022
7	-0.01	0.05	-0.107998	0.087998	13 February 2022 to 26 February 2022
8	-0.1	0.04	-0.178399	-0.021601	27 February 2022 to 12 March 2022
9	-0.04	0.03	-0.098799	0.018799	13 March 2022 to 26 March 2022
10	-0.14	0.03	-0.198799	-0.081201	27 March 2022 to 09 April 2022
11	-0.02	0.04	-0.098399	0.058399	10 April 2022 to 23 April 2022
12	0.06	0.05	-0.037998	0.157998	24 April 2022 to 07 May 2022

Non-combinability of studies

Cochran Q = 27.329148 (df = 11) P = 0.0041
Moment-based estimate of between studies variance = 0.002715
I_2 (inconsistency) = 59.7% (95% CI = 6.7% to 77.1%)

Random effects (DerSimonian-Laird)

Pooled risk difference = -0.075461 (95% CI = -0.11487 to -0.036051)
Z (test Risk Difference) = -3.752923 P = 0.0002

Summary meta-analysis plot [random effects]

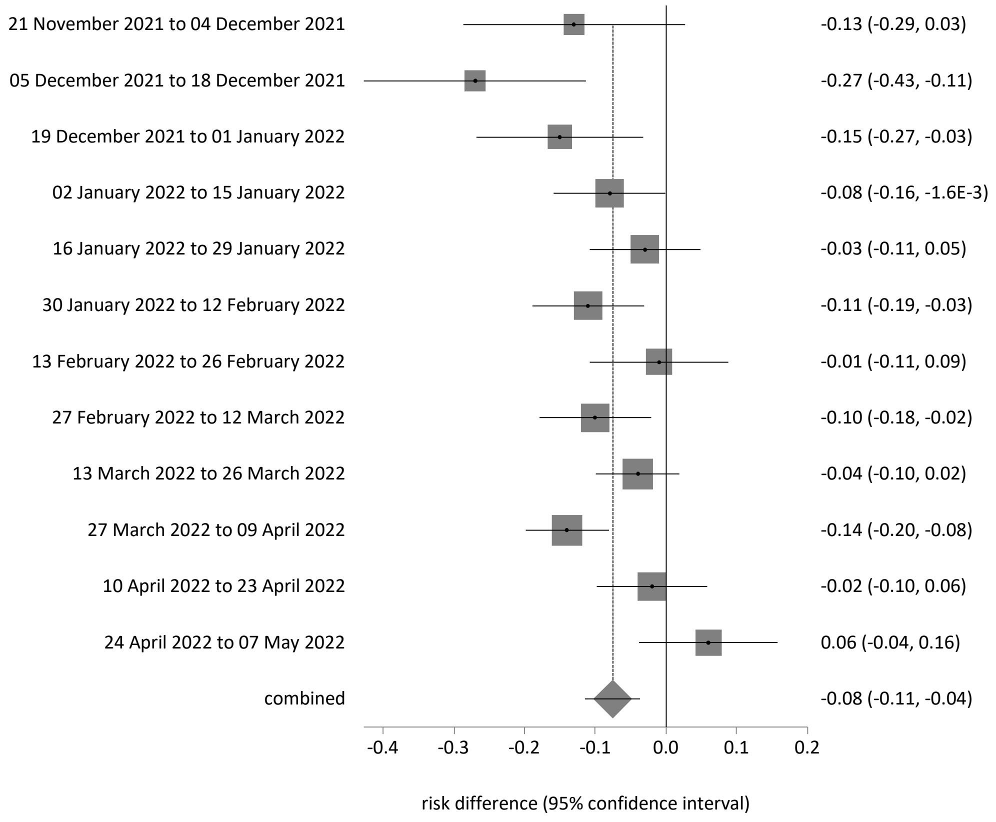

Total physical contacts in the last 7 days 1 to 10 v none

Time period	Risk Difference	SE	Approximate 95% CI		
1	0.12	0.06	0.002402	0.237598	21 November 2021 to 04 December 2021
2	0.12	0.06	0.002402	0.237598	05 December 2021 to 18 December 2021
3	0.12	0.04	0.041601	0.198399	19 December 2021 to 01 January 2022
4	0.1	0.03	0.041201	0.158799	02 January 2022 to 15 January 2022
5	0.22	0.04	0.141601	0.298399	16 January 2022 to 29 January 2022
6	0.24	0.03	0.181201	0.298799	30 January 2022 to 12 February 2022
7	0.18	0.04	0.101601	0.258399	13 February 2022 to 26 February 2022
8	0.13	0.03	0.071201	0.188799	27 February 2022 to 12 March 2022
9	0.13	0.03	0.071201	0.188799	13 March 2022 to 26 March 2022
10	0.15	0.03	0.091201	0.208799	27 March 2022 to 09 April 2022
11	0.19	0.03	0.131201	0.248799	10 April 2022 to 23 April 2022
12	0.12	0.05	0.022002	0.217998	24 April 2022 to 07 May 2022

Non-combinability of studies

Cochran Q = 19.118829 (df = 11) P = 0.059
Moment-based estimate of between studies variance = 0.000948
I_2 (inconsistency) = 42.5% (95% CI = 0% to 69.3%)

Random effects (DerSimonian-Laird)

Pooled risk difference = 0.155282 (95% CI = 0.127978 to 0.182585)
Z (test Risk Difference) = 11.146846 P < 0.0001

Summary meta-analysis plot [random effects]

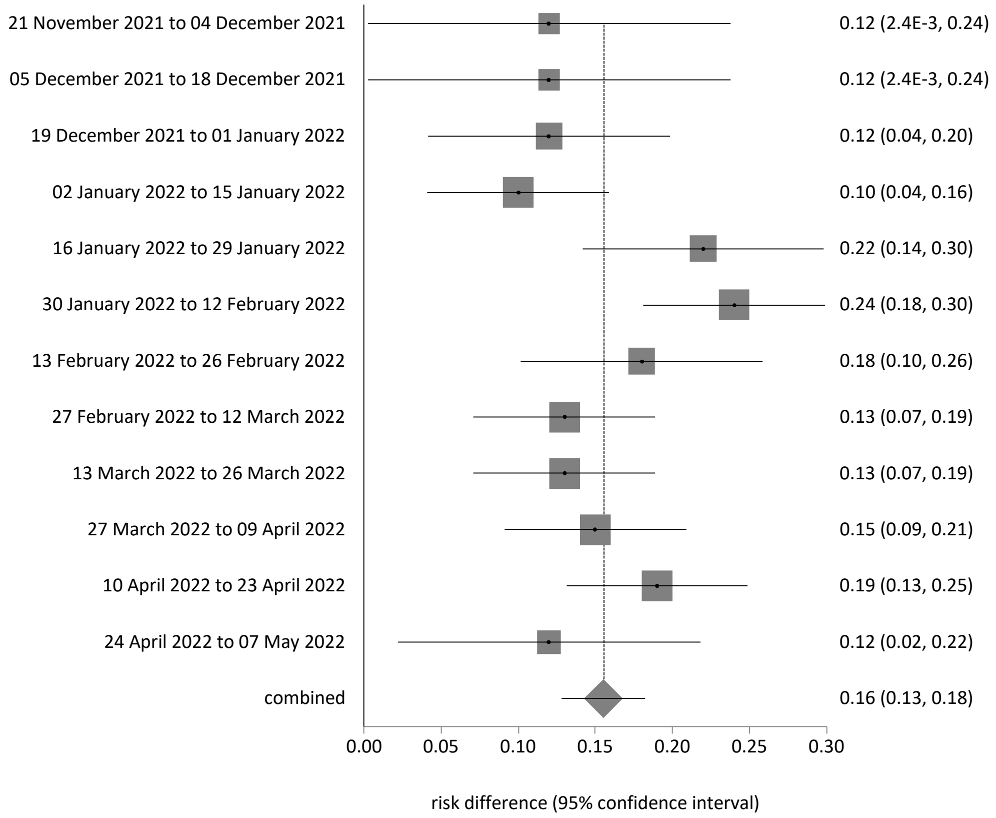

Total physical contacts in the last 7 days 11 to 20 v none

Time period	Risk Difference	SE	Approximate 95% CI		
1	0.12	0.08	-0.036797	0.276797	21 November 2021 to 04 December 2021
2	0.21	0.07	0.072803	0.347197	05 December 2021 to 18 December 2021
3	0.31	0.06	0.192402	0.427598	19 December 2021 to 01 January 2022
4	0.25	0.04	0.171601	0.328399	02 January 2022 to 15 January 2022
5	0.35	0.05	0.252002	0.447998	16 January 2022 to 29 January 2022
6	0.28	0.04	0.201601	0.358399	30 January 2022 to 12 February 2022
7	0.32	0.05	0.222002	0.417998	13 February 2022 to 26 February 2022
8	0.27	0.05	0.172002	0.367998	27 February 2022 to 12 March 2022
9	0.22	0.04	0.141601	0.298399	13 March 2022 to 26 March 2022
10	0.21	0.04	0.131601	0.288399	27 March 2022 to 09 April 2022
11	0.25	0.05	0.152002	0.347998	10 April 2022 to 23 April 2022
12	0.19	0.07	0.052803	0.327197	24 April 2022 to 07 May 2022

Non-combinability of studies

Cochran Q = 12.799933 (df = 11) P = 0.3066
Moment-based estimate of between studies variance = 0.000404
I_2 (inconsistency) = 14.1% (95% CI = 0% to 56.6%)

Random effects (DerSimonian-Laird)

Pooled risk difference = 0.255423 (95% CI = 0.224991 to 0.285856)
Z (test Risk Difference) = 16.450107 P < 0.0001

Summary meta-analysis plot [random effects]

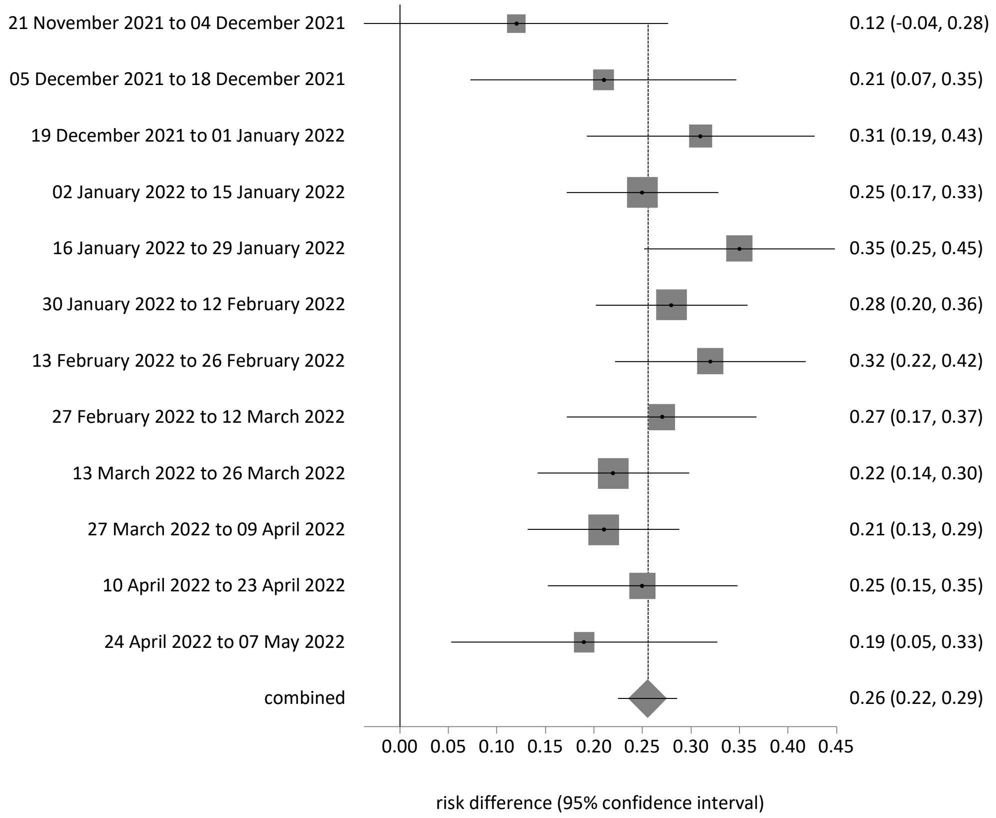

Total physical contacts in the last 7 days 21 to 40 v none

Time period	Risk Difference	SE	Approximate 95% CI		
1	0.1	0.08	-0.056797	0.256797	21 November 2021 to 04 December 2021
2	0.3	0.07	0.162803	0.437197	05 December 2021 to 18 December 2021
3	0.27	0.06	0.152402	0.387598	19 December 2021 to 01 January 2022
4	0.24	0.04	0.161601	0.318399	02 January 2022 to 15 January 2022
5	0.33	0.05	0.232002	0.427998	16 January 2022 to 29 January 2022
6	0.25	0.05	0.152002	0.347998	30 January 2022 to 12 February 2022
7	0.39	0.05	0.292002	0.487998	13 February 2022 to 26 February 2022
8	0.31	0.05	0.212002	0.407998	27 February 2022 to 12 March 2022
9	0.31	0.04	0.231601	0.388399	13 March 2022 to 26 March 2022
10	0.17	0.04	0.091601	0.248399	27 March 2022 to 09 April 2022
11	0.15	0.05	0.052002	0.247998	10 April 2022 to 23 April 2022
12	0.14	0.08	-0.016797	0.296797	24 April 2022 to 07 May 2022

Non-combinability of studies

Cochran Q = 28.007517 (df = 11) P = 0.0032
Moment-based estimate of between studies variance = 0.004039
I_2 (inconsistency) = 60.7% (95% CI = 9.8% to 77.6%)

Random effects (DerSimonian-Laird)

Pooled risk difference = 0.253184 (95% CI = 0.206077 to 0.300292)
Z (test Risk Difference) = 10.53412 P < 0.0001

Summary meta-analysis plot [random effects]

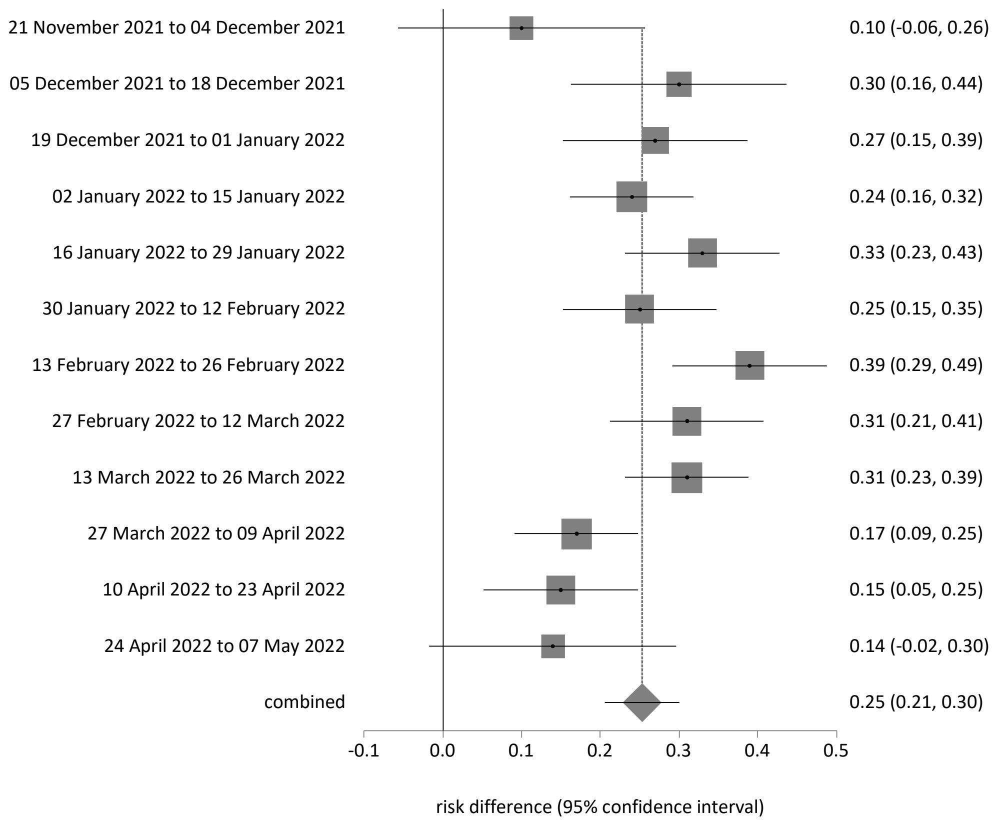

Total physical contacts in the last 7 days 40+ v none

Time period	Risk Difference	SE	Approximate 95% CI		
1	0.11	0.14	-0.164395	0.384395	21 November 2021 to 04 December 2021
2	0.2	0.12	-0.035196	0.435196	05 December 2021 to 18 December 2021
3	0.3	0.09	0.123603	0.476397	19 December 2021 to 01 January 2022
4	0.35	0.07	0.212803	0.487197	02 January 2022 to 15 January 2022
5	0.28	0.09	0.103603	0.456397	16 January 2022 to 29 January 2022
6	0.04	0.1	-0.155996	0.235996	30 January 2022 to 12 February 2022
7	0.35	0.1	0.154004	0.545996	13 February 2022 to 26 February 2022
8	0.22	0.09	0.043603	0.396397	27 February 2022 to 12 March 2022
9	0.19	0.07	0.052803	0.327197	13 March 2022 to 26 March 2022
10	0.23	0.07	0.092803	0.367197	27 March 2022 to 09 April 2022
11	0.21	0.09	0.033603	0.386397	10 April 2022 to 23 April 2022
12	0.21	0.13	-0.044795	0.464795	24 April 2022 to 07 May 2022

Non-combinability of studies

Cochran Q = 10.029316 (df = 11) P = 0.5278
Moment-based estimate of between studies variance = 0
I_2 (inconsistency) = 0% (95% CI = 0% to 49.8%)

Random effects (DerSimonian-Laird)

Pooled risk difference = 0.237436 (95% CI = 0.186588 to 0.288285)
Z (test Risk Difference) = 9.151963 P < 0.0001

Summary meta-analysis plot [random effects]

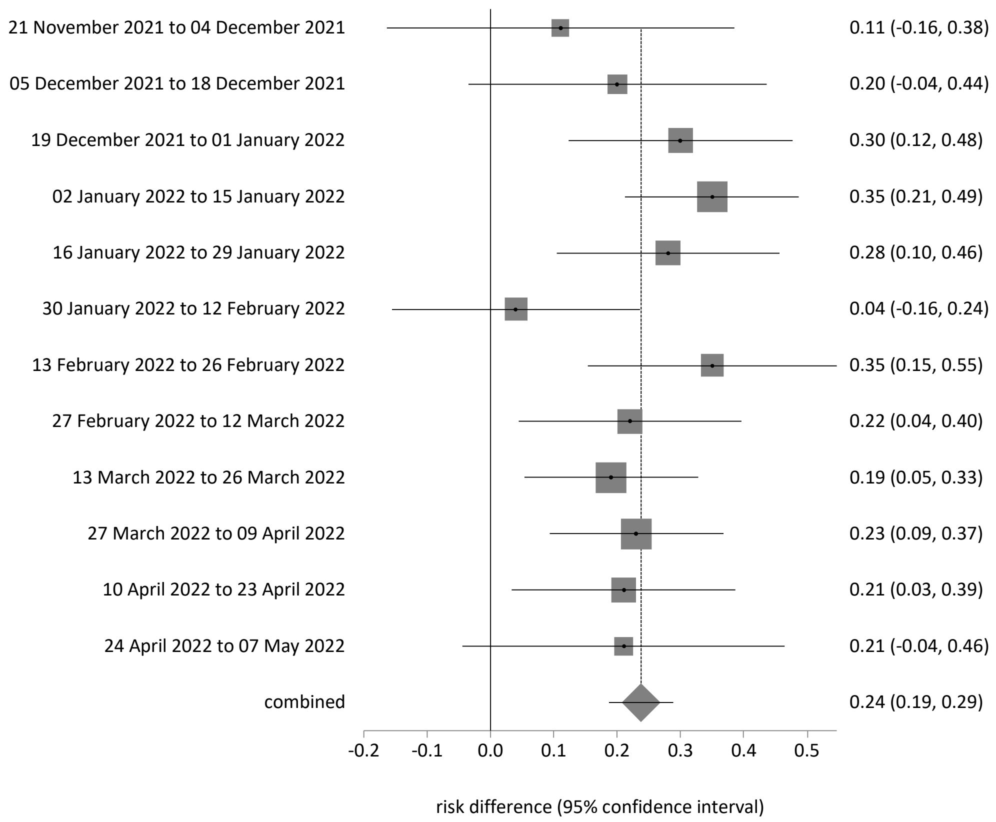

Total socially distanced contacts in the last 7 days 1 to 10 v none

Time period	Risk Difference	SE	Approximate 95% CI		
1	0.01	0.08	-0.146797	0.166797	21 November 2021 to 04 December 2021
2	0.06	0.08	-0.096797	0.216797	05 December 2021 to 18 December 2021
3	-0.02	0.06	-0.137598	0.097598	19 December 2021 to 01 January 2022
4	-0.08	0.04	-0.158399	-0.001601	02 January 2022 to 15 January 2022
5	0	0.04	-0.078399	0.078399	16 January 2022 to 29 January 2022
6	0.12	0.04	0.041601	0.198399	30 January 2022 to 12 February 2022
7	0.09	0.05	-0.007998	0.187998	13 February 2022 to 26 February 2022
8	0.03	0.04	-0.048399	0.108399	27 February 2022 to 12 March 2022
9	0.09	0.03	0.031201	0.148799	13 March 2022 to 26 March 2022
10	0.09	0.03	0.031201	0.148799	27 March 2022 to 09 April 2022
11	0.05	0.04	-0.028399	0.128399	10 April 2022 to 23 April 2022
12	0.03	0.06	-0.087598	0.147598	24 April 2022 to 07 May 2022

Non-combinability of studies

Cochran Q = 21.365372 (df = 11) P = 0.0298
Moment-based estimate of between studies variance = 0.001759
I_2 (inconsistency) = 48.5% (95% CI = 0% to 72%)

Random effects (DerSimonian-Laird)

Pooled risk difference = 0.043634 (95% CI = 0.008395 to 0.078872)
Z (test Risk Difference) = 2.426921 P = 0.0152

Summary meta-analysis plot [random effects]

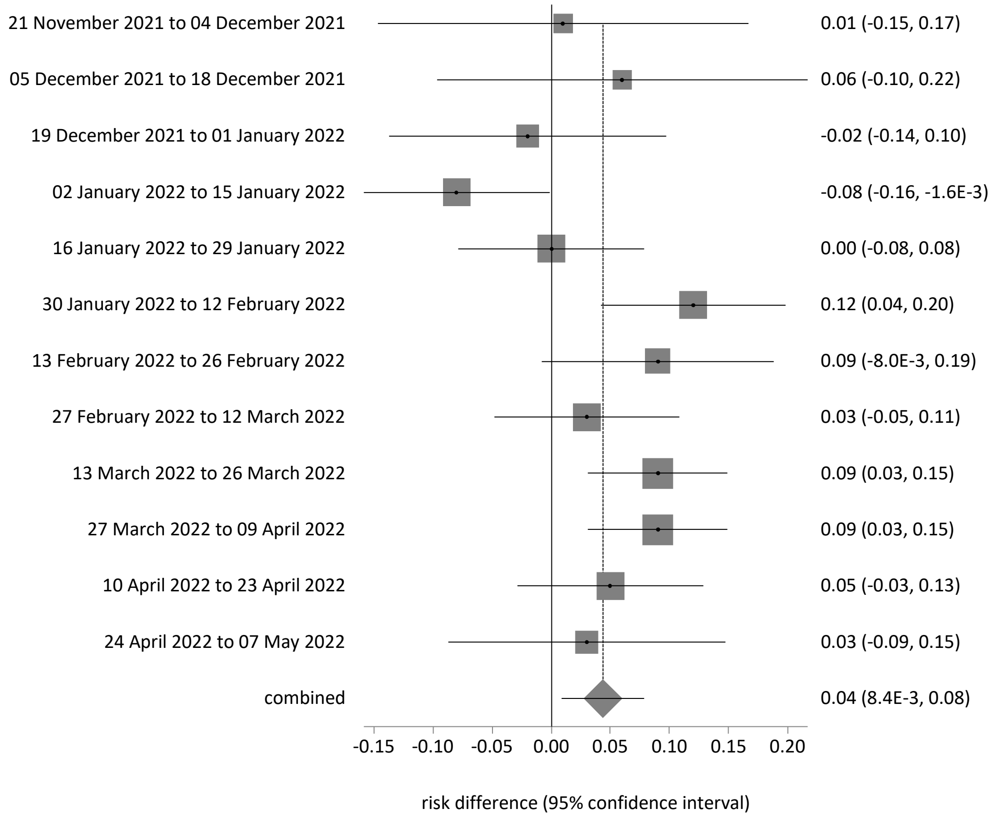

Total socially distanced contacts in the last 7 days 11 to 20 v none

Time period	Risk Difference	SE	Approximate 95% CI		
1	0.1	0.09	-0.076397	0.276397	21 November 2021 to 04 December 2021
2	0.22	0.09	0.043603	0.396397	05 December 2021 to 18 December 2021
3	0.15	0.06	0.032402	0.267598	19 December 2021 to 01 January 2022
4	0.01	0.05	-0.087998	0.107998	02 January 2022 to 15 January 2022
5	0.05	0.05	-0.047998	0.147998	16 January 2022 to 29 January 2022
6	0.18	0.05	0.082002	0.277998	30 January 2022 to 12 February 2022
7	0.24	0.06	0.122402	0.357598	13 February 2022 to 26 February 2022
8	0.21	0.05	0.112002	0.307998	27 February 2022 to 12 March 2022
9	0.25	0.04	0.171601	0.328399	13 March 2022 to 26 March 2022
10	0.2	0.04	0.121601	0.278399	27 March 2022 to 09 April 2022
11	0.15	0.05	0.052002	0.247998	10 April 2022 to 23 April 2022
12	0.15	0.07	0.012803	0.287197	24 April 2022 to 07 May 2022

Non-combinability of studies

Cochran Q = 23.735606 (df = 11) P = 0.0139
Moment-based estimate of between studies variance = 0.003324
I_2 (inconsistency) = 53.7% (95% CI = 0% to 74.3%)

Random effects (DerSimonian-Laird)

Pooled risk difference = 0.160516 (95% CI = 0.114952 to 0.20608)
Z (test Risk Difference) = 6.90466 P < 0.0001

Summary meta-analysis plot [random effects]

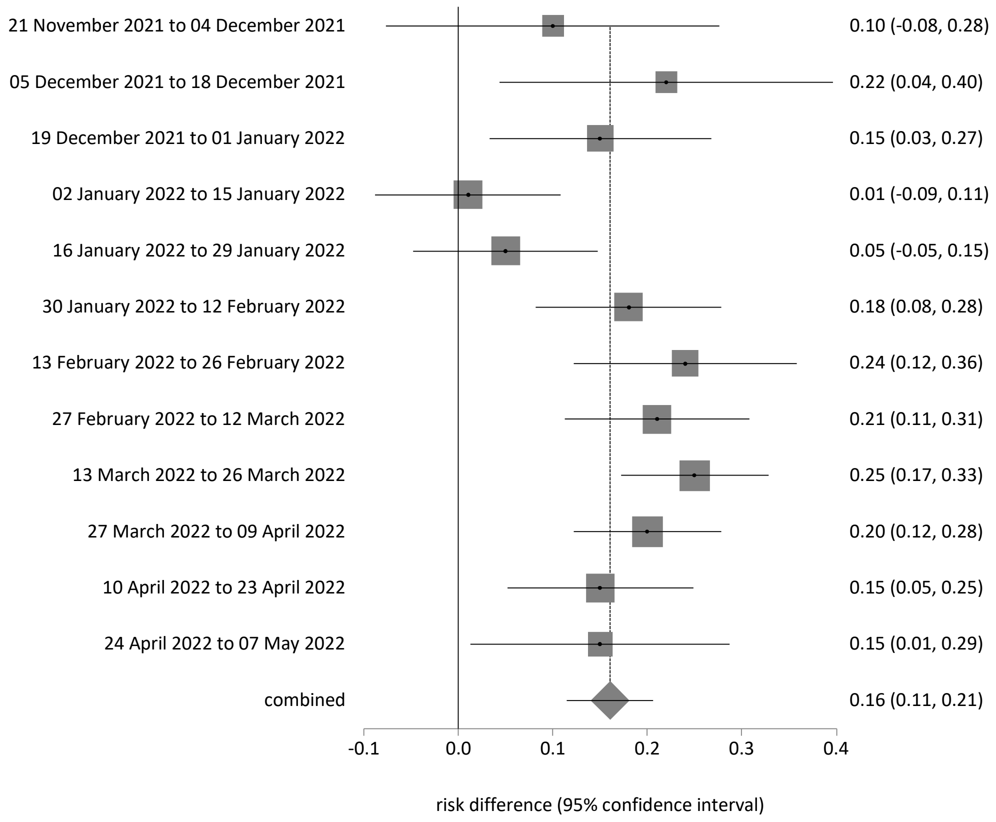

Total socially distanced contacts in the last 7 days 21 to 40 v none

Time period	Risk Difference	SE	Approximate 95% CI		
1	0.19	0.08	0.033203	0.346797	21 November 2021 to 04 December 2021
2	0.28	0.08	0.123203	0.436797	05 December 2021 to 18 December 2021
3	0.16	0.06	0.042402	0.277598	19 December 2021 to 01 January 2022
4	0.1	0.04	0.021601	0.178399	02 January 2022 to 15 January 2022
5	0.1	0.04	0.021601	0.178399	16 January 2022 to 29 January 2022
6	0.19	0.04	0.111601	0.268399	30 January 2022 to 12 February 2022
7	0.31	0.05	0.212002	0.407998	13 February 2022 to 26 February 2022
8	0.23	0.05	0.132002	0.327998	27 February 2022 to 12 March 2022
9	0.29	0.04	0.211601	0.368399	13 March 2022 to 26 March 2022
10	0.24	0.04	0.161601	0.318399	27 March 2022 to 09 April 2022
11	0.21	0.05	0.112002	0.307998	10 April 2022 to 23 April 2022
12	0.24	0.07	0.102803	0.377197	24 April 2022 to 07 May 2022

Non-combinability of studies

Cochran Q = 25.588384 (df = 11) P = 0.0075
Moment-based estimate of between studies variance = 0.003164
I_2 (inconsistency) = 57% (95% CI = 0% to 75.9%)

Random effects (DerSimonian-Laird)

Pooled risk difference = 0.207793 (95% CI = 0.164614 to 0.250973)
Z (test Risk Difference) = 9.431894 P < 0.0001

Summary meta-analysis plot [random effects]

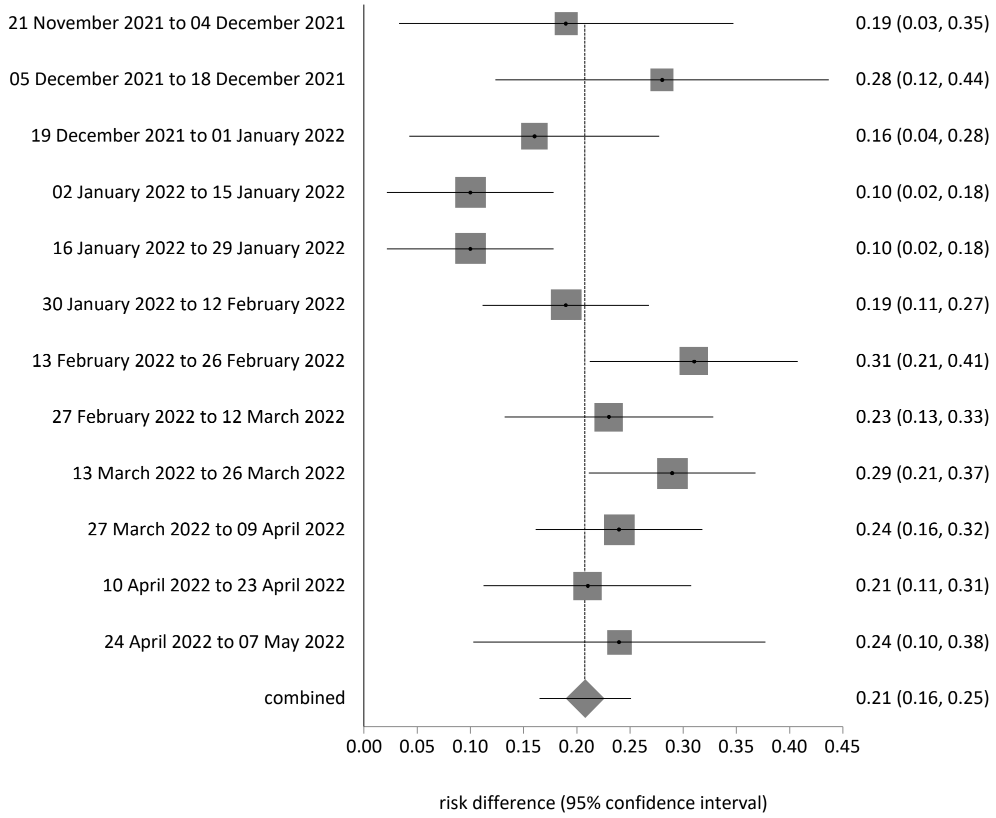

Total socially distanced contacts in the last 7 days   >40 v none

Time period	Risk Difference	SE	Approximate 95% CI		
1	0.21	0.09	0.033603	0.386397	21 November 2021 to 04 December 2021
2	0.27	0.08	0.113203	0.426797	05 December 2021 to 18 December 2021
3	0.17	0.06	0.052402	0.287598	19 December 2021 to 01 January 2022
4	0.03	0.05	-0.067998	0.127998	02 January 2022 to 15 January 2022
5	0.11	0.05	0.012002	0.207998	16 January 2022 to 29 January 2022
6	0.22	0.05	0.122002	0.317998	30 January 2022 to 12 February 2022
7	0.24	0.06	0.122402	0.357598	13 February 2022 to 26 February 2022
8	0.2	0.05	0.102002	0.297998	27 February 2022 to 12 March 2022
9	0.31	0.04	0.231601	0.388399	13 March 2022 to 26 March 2022
10	0.23	0.04	0.151601	0.308399	27 March 2022 to 09 April 2022
11	0.22	0.05	0.122002	0.317998	10 April 2022 to 23 April 2022
12	0.25	0.07	0.112803	0.387197	24 April 2022 to 07 May 2022

Non-combinability of studies

Cochran Q = 25.085915   (df = 11) P = 0.0089
Moment-based estimate of between studies variance = 0.003644
I_2 (inconsistency) = 56.2% (95% CI = 0% to 75.5%)

Random effects (DerSimonian-Laird)

Pooled risk difference = 0.203352 (95% CI = 0.156806 to 0.249898)
Z (test Risk Difference) = 8.562786   P < 0.0001

Summary meta-analysis plot [random effects]

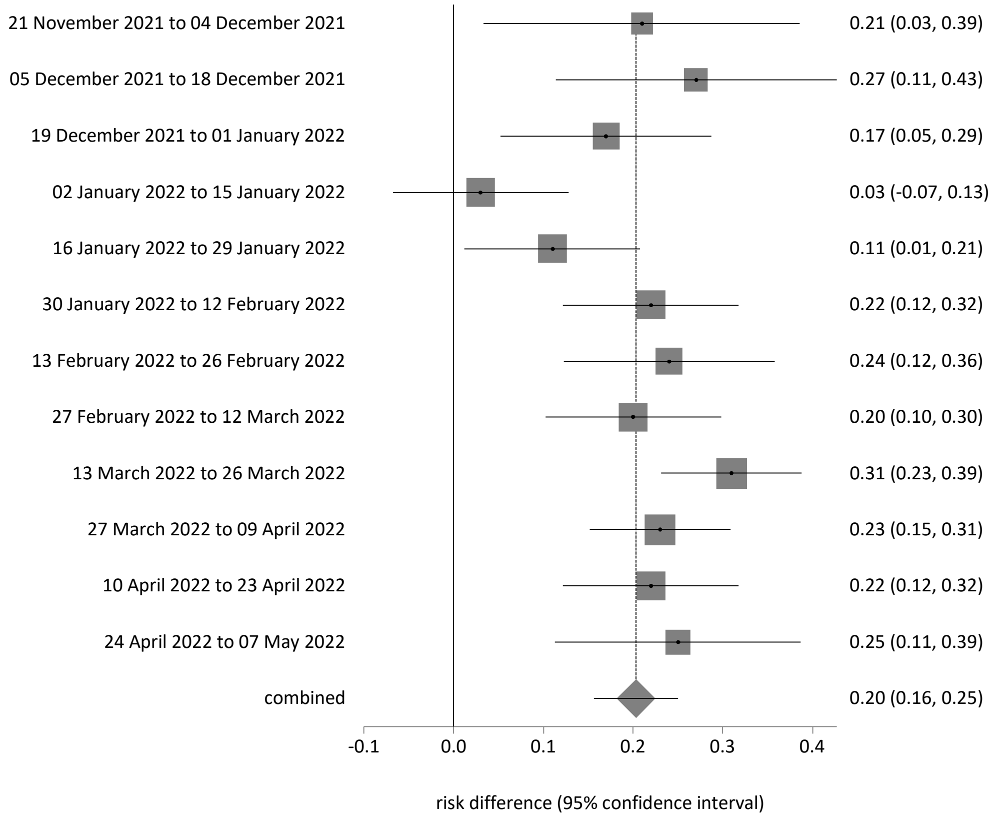

Supplement: S1 Appendix — (PDF) [file pone.0299714.s001.pdf]
