# Supplementary material for: Changing risk factors for developing SARS-CoV-2 infection from Delta to Omicron
Source: PLoS One. 2024 May 15;19(5):e0299714. doi: 10.1371/journal.pone.0299714 (PMC11095668; doi:10.1371/journal.pone.0299714)
Supplement: Attachment — Submitted filename: R2R.docx [file pone.0299714.s002.docx]

Thanks to referees for helpful comments.

Editors: Please also see separate cover letter about editorial revisions.

| **Comment** | | | | **Response** | |
| --- | --- | --- | --- | --- | --- |
| ***Editor(s)*** | | | |  | |
| - A rebuttal letter that responds to each point raised by the academic editor and reviewer(s). You should upload this letter as a separate file labeled 'Response to Reviewers'. - A marked-up copy of your manuscript that highlights changes made to the original version. You should upload this as a separate file labeled 'Revised Manuscript with Track Changes'. - An unmarked version of your revised paper without tracked changes. You should upload this as a separate file labeled 'Manuscript'. | | | | | These items are each provided |
| Please ensure that your manuscript meets PLOS ONE's style requirements, including those for file naming. | | | | We believe that this requirement is complied with | |
| Please state what role the funders took in the study.  If the funders had no role, please state: "The funders had no role in study design, data collection and analysis, decision to publish, or preparation of the manuscript." | | | | "The funders had no role in study design, data collection and analysis, decision to publish, or preparation of the manuscript." | |
| While revising your submission, please upload your figure files to the Preflight Analysis and Conversion Engine (PACE) digital diagnostic tool, (URL supplied) | | | | The figures were checked on PACE and seem to be compliant. | |
| ***Reviewer #1*** | | |  | | |
| 1. The method section in the Abstract is too short. Please add more information on the data and the statistical analyses. | | | Information was added about the data and methods. | | |
| The estimates should be reported in the result section. [of abstract] | | We declined to do this because it is so complicated to summarise. There weren’t just one or two estimates, there are many dozens of numbers that we could report, especially if the lack of change (null relationship) results are just as important as the items that did change risk of positivity (and why wouldn’t they just be as important to mention).  We did add text to the results to emphasise that some risk factors did not change. | | | |
| The conclusions should be the implications and interpretation of the results. | We believe that we have included many comments on implications and interpretation. The format of PlosOne articles is that there is no entitled Conclusions section. | | | | |
| 2. I suggest replacing the term “likelihood of testing positive” with the risk/likelihood of infection. | The phrase “likelihood of testing positive” is the one used by Office of National Statistics from which we took the data. If we start using terms different to what ONS published the data under, it would cause confusion to anyone trying to replicate our work. The term is also more technically correct due to the fact that some results of testing would be false positive and some false negative. | | | | |
| 3. As the study period across phases where different variants are circulating, a variable related to these distinct phases should be included in the meta-regression model instead of the period number, such that the interpretation of the estimates can be compared among different variants' predominant phases. | We did consider this but as we stated in the methods section, we were basically handling summary analysis data produced by ONS and that was grouped by fortnight. For several of the time periods there were multiple variants circulating that varied even from the beginning to end of the two-week period. Thus, making modelling of the variant difficult. Identifying the dominant variant complex. Also, as we discussed in the discussion one of the more likely explanations of these findings was due not so much to the variant but to the impact of changing population immunity as described by the SEIRS model. So using variant as the predictor would pre-suppose the mechanism of the effect and also hide the impact of change that occurred during the ascendancy of a single variant. However, we have modified the main figures to include a graphic illustration the relative proportion of the different variants during the study periods | | | | |
| 4. Given that the meta-regression was often used in the context of substantial between-study variations, the metric I2 should be reported for each pooled estimate. | | “I^2^, provides an estimate of the proportion of variability in a meta-analysis that is explained by differences between the included trials rather than by sampling error”. Also “I^2^ estimates need to be interpreted with caution when the meta-analysis only includes a limited number of events or trials.”. By limited number of studies the statistical papers suggest less than 15.  [https://journals.plos.org/plosone/ article?id=10.1371/journal.pone.0039471](https://journals.plos.org/plosone/%20article?id=10.1371/journal.pone.0039471)  So given the fact that our analyses were of a single study that was repeated and not multiple studies we would argue that the I^2^ test was not appropriate. Instead, we were primarily interested in whether there was a trend over time and for that analysis t test for trend over time was the most appropriate metric. Nevertheless, for those predictor variables where there was not a significant trend we now include I^2^ and Cochranes Q. Also, we have now described in more detail the data and methods used and given a justification in the discussion | | | |
| 5. Please be consistent about the term “Covid-19” (e.g., instead of “covid”) throughout the text. | | Done (all changed to COVID-19) | | | |
| 6. Why are the results for multigeneration households reversed compared to household sizes? Please state the difference between multigeneration household and normal household. | | We understand referee to be saying that if larger households have a trend (increasing with household size) of elevated risk of infection, why did multi-generational households have lower risk of infection.  We suggest in the discussion that this result suggests that multi-generational households are not reliably also the largest households. We cannot explore the relationship further with the data available.  A definition with reference was added, per below information.  The UK Census as undertaken by ONS defines a mutigenerational household as “Households where people from across more than two generations of the same family live together. This includes households with grandparents and grandchildren whether or not the intervening generation also live in the household.” https://www.ons.gov.uk/census/census2021dictionary/ variablesbytopic/demographyvariablescensus2021/multiplegenerationsinhousehold | | | |
| 7. Please change Table 1 to a figure similar to figure 1-4 but combining all the pooled estimates shown in Table 1. | | Not done.  If we did this we would not be able to include the T test for trend which was the primary aim of this study to see whether risk factors changed over time in the same population. | | | |
| 8. It is unclear why the authors only show the detailed estimates during the periods for 4 strata in table1. I suggest generating the same figure for all other strata as well and moving all these figures to the Appendix. | | We do not understand this comment as there were no different strata in this analysis. These were categorical predictor variables. If we had access to the primary data, we would simply have looked for trend as an interaction term with each predictor. What we have done in this study is mimic as closely as we can the analyses that would have been done if we had the primary data. However, we have now added analyses to all predictor variables whether or not they showed a significant trend and placed these in the supplementary file. | | | |
| 9. The strata “Adults, living in a household with someone aged 16 or under” and “Aged under 70 years, living in a household with someone aged 70 or over ” are different from the figure2 legend. | | We revised the figure legend and some of the narrative text | | | |
| 10. Please use add the full form of “effective R”. | | Done | | | |
| ***Reviewer #2*** | |  | | | |
| It is unclear to me why the authors did not include some of the statistically significant predictor variables that they reported in Table 1 (such as Traveling abroad, Rurality, and Employment status) in their subsequent analysis. | | The paper was primarily about whether risk factors change over time, ie whether there is a trend. We only presented the forest plots to illustrate the temporal change where there was a significant t test for trend. Where there was no trend then any differences between time periods would only represent random variation between times and so would detract from the primary purpose of the study. We have however, now added all analyses not highlighted in the main paper to a supplementary file.  We have also explained better our methods, that the data are based on a single survey, and provided additional justification for the meta-regression approach in the Discussion. | | | |
| Page 3: “We report a study to determine whether risk factors for infection after the emergence of the omicron variant.”. Please rephrase for clarity. | | Inserted ‘changed’ between infection and after | | | |
| Please replace “covid” with “COVID-19” across the manuscript when discussing and referring to COVID-19. | | Done | | | |
| Table 1: Please highlight statistically significant values using the asterisk sign and define the p-value cutoff in the table's footnote. | | Done | | | |
